# Ultrafine Molybdenum Wire Braided Neurointerventional Implants: Bridging Biodegradability and Neurovascular Safety for Stroke Treatment

**DOI:** 10.1002/advs.202511466

**Published:** 2025-09-29

**Authors:** Yunong Shen, Yiming Huang, Yang Zhang, Chunhao Yu, Hanqi Liu, Dong Bian, Di Wu, Wu Wang, Xunming Ji, Yufeng Zheng, Miaowen Jiang, Ming Li

**Affiliations:** ^1^ School of Materials Science and Engineering Peking University Beijing 100871 China; ^2^ China‐America Institute of Neuroscience of Xuanwu Hospital Beijing Institute for Brain Disorders Capital Medical University Beijing 100053 China; ^3^ Department of Neurology Xuanwu Hospital Capital Medical University Beijing 100053 China; ^4^ Department of Neurosurgery Capital Medical University Beijing 100053 China; ^5^ School of Life Beijing Institute of Technology Beijing 100081 China; ^6^ Medical Research Institute Department of Orthopedics Guangdong Provincial People's Hospital (Guangdong Academy of Medical Sciences) Southern Medical University Guangzhou 510080 China; ^7^ Department of Radiology Longhua Hospital Shanghai University of Traditional Chinese Medicine Shanghai 200032 China

**Keywords:** aneurysm coils, in vivo degradation, molybdenum, neuro compatibility, stroke stent

## Abstract

Neurovascular implants for stroke intervention face a critical dilemma: permanent devices (e.g., nitinol stents, platinum coils) often trigger chronic inflammation and recurrence, whereas biodegradable alternatives (Mg, Fe, Zn alloys) lack radiopacity or raise neurotoxicity concerns. Here, we introduce φ50 µm molybdenum (Mo) wire braided implants that integrate procedural efficacy with biological safety. Mo demonstrates negligible hemolysis (<5%), platelet‐inert surfaces, and preserved coagulation kinetics, together with robust cytocompatibility across neurovascular unit cells (endothelia, astrocytes, neurons) under both physiological and ischemia–reperfusion conditions. In vivo, Mo stent wires implanted in rodent carotids maintained blood homeostasis, organ integrity, and neurological function without systemic toxicity. Moreover, braided 2D Mo coils achieved durable aneurysm occlusion with controlled inflammatory resolution and progressive endothelialization, closely resembling clinical performance. Importantly, Mo ions showed no detectable accumulation in brain, kidney, lung, or spleen, attributable to renal clearance and blood–brain barrier selectivity. By coupling intrinsic radiopacity with homogeneous, moderate corrosion, Mo addresses long‐standing limitations of existing biodegradable alloys. These findings position Mo as a transformative candidate for next‐generation neurovascular devices, harmonizing biodegradability, safety, and imaging precision to redefine the management of both ischemic and hemorrhagic stroke.

## Graphical abstract

Molybdenum (Mo), with its unique strength, uniform corrosion, and radiopacity, enables innovative biodegradable implants for transformative stroke therapy.

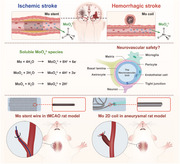

## 1 Introduction

Stroke remains the second leading cause of global mortality and the third leading cause of world disability.^[^ ^1^ ^]^ Its intervention treatment faces a dual therapeutic challenge for the scenario of ischemic strokes require stent‐assisted angioplasty^[^ ^2^ ^]^ and hemorrhagic cases necessitate aneurysm coiling.^[^ ^3^ ^]^ Current permanent nondegradable implants, though life‐saving, create long‐term biocompatibility dilemmas. For ischemic stroke treatment, nitinol stents and flow diverters induce higher in‐stent restenosis within 2 years due to chronic inflammation.^[^ ^4^ ^]^ In hemorrhagic management, the platinum coil exhibits high postoperative recurrence rate of aneurysms at 18 months.^[^ ^5^ ^]^ These dual clinical demands necessitate advanced implant technologies addressing both ischemic and hemorrhagic sequelae.

The shift from nondegradable metal implants to degradable ones could be a promising approach to tack with those issues. Current biodegradable strategies predominantly focuses on magnesium (Mg), iron (Fe), and zinc (Zn)‐based alloys yet face limitations.^[^ ^6^ ^]^ Mg alloys have drawbacks, such as rapid degradation, severe pitting corrosion, and a high risk of stent fracture.^[^ ^7^ ^]^ Zn alloys exhibit characteristic like neurotoxicity, aging at room temperature, and poor creep resistance.^[^ ^8^, ^9^ ^]^ Fe alloys degrade slowly and have ferromagnetic properties, which lead to incompatibility with MRI.^[^ ^10^ ^]^ These shortcomings of those biodegradable metals make them unsuitable for the design of biodegradable neuro‐interventional devices.

**Figure 1:**
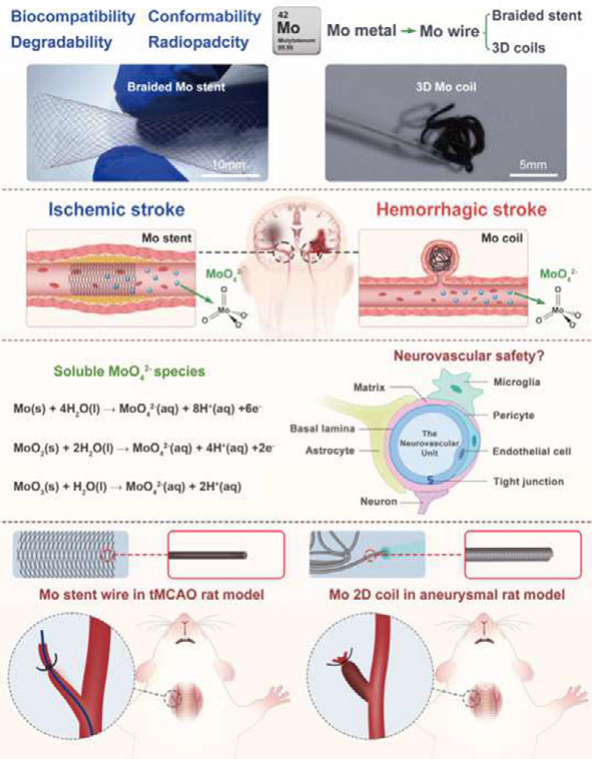
Schematic illustration of the experimental design. A) Application of Mo wire braided stents and coils in treating intracranial ischemic and hemorrhagic strokes. B) Photographs of Mo braided stent wire and 3D aneurysm coils. C) In vivo degradation process of Mo implants and composition of the neurovascular unit (NVU). D) Schematic workflow for evaluating the histopathological and functional safety of Mo braided stent wire and 2D coils in rat models of ischemic stroke and cerebral aneurysms.

Molybdenum (Mo) emerges as a competitive biodegradable metal with distinctive physicochemical properties for neuro‐interventional applications (**Figure** **1**A). It can be processed into mechanically‐ultrafine wire (φ50 µm), which enables the fabrication of “thin‐walled” braided stents (<80 µm strut thickness) with high possibility of enhanced radial compliance and accelerated endothelial coverage, and the manufacture of aneurysm coils with high potential of good flexibility and reduced mechanical stress on fragile aneurysm walls. Besides, Mo exhibits homogeneous degradation kinetics,^[^ ^11^ ^]^ preventing stress‐induced fractures observed in other biodegradable metal stents due to localized pitting corrosion that compromises mechanical integrity. Second, Mo has superior density, enables fabrication of intracranial stents/coils with enhanced radiopacity for precise device deployment in tortuous cerebrovasculature.^[^ ^12^ ^]^ Third, Mo has been investigated as brain biosensing,^[^ ^13^, ^14^, ^15^, ^16^ ^]^ cardiovascular stents,^[^ ^17^ ^]^ and bone repair implants^[^ ^18^ ^]^ with promising biocompatibilities. These attributes position Mo as a promising candidate for next‐generation neurovascular implants, such as cerebral stent and coil (Figure 1B; and Figures S1 and S2, Supporting Information).

The neurocompatibility evaluation of biodegradable metals requires specialized consideration given the central nervous system's heightened sensitivity to ionic species.^[^ ^6^, ^19^ ^]^ And the released ions can be transferred into the brain through blood circulation.^[^ ^20^, ^21^ ^]^ Clinical evidence reveals that most of stroke patients exhibit compromised blood–brain barrier (BBB) integrity,^[^ ^22^ ^]^ potentially amplifying neurotoxicity risks from metallic degradation products.^[^ ^19^ ^]^ This is particularly critical for making innovative neurointerventional devices. Although existing studies have reported the biosafety of Mo as endovascular devices^[^ ^23^ ^]^ and even for cerebral applications,^[^ ^13^, ^14^ ^]^ most of these evaluations were conducted using healthy cells and animal models. These approaches fail to simulate the pathological microenvironment of actual disease conditions. Therefore, systematic investigation into the neurocompatibility of Mo remains imperative, particularly under disease‐relevant physiological conditions.

In this study, we first evaluated the effects of degradation products of pure Mo on large artery related cells (including endothelial cells (ECs) and smooth muscle cells (SMCs)) and healthy or injured neurovascular unit (NVU) cells (including microvessel endothelial cells (bEnd.3), astrocytes (C8‐D1A), and neurons (HT22)) in vitro (Figure 1C). Then, Mo braided stent wire and coils were implanted in common carotid artery (CCA) of transient middle cerebral artery occlusion (tMCAO) rat model and external carotid artery (ECA) of aneurysm rat model, respectively, to evaluate its whole‐body biosafety as biodegradable neurointerventional device metals in potential treatment on acute ischemic stroke and hemorrhagic stroke (Figure 1D).

## 2 Results

### 2.1 In Vitro Hemocompatibility of Mo Wire

**Figure 2:**
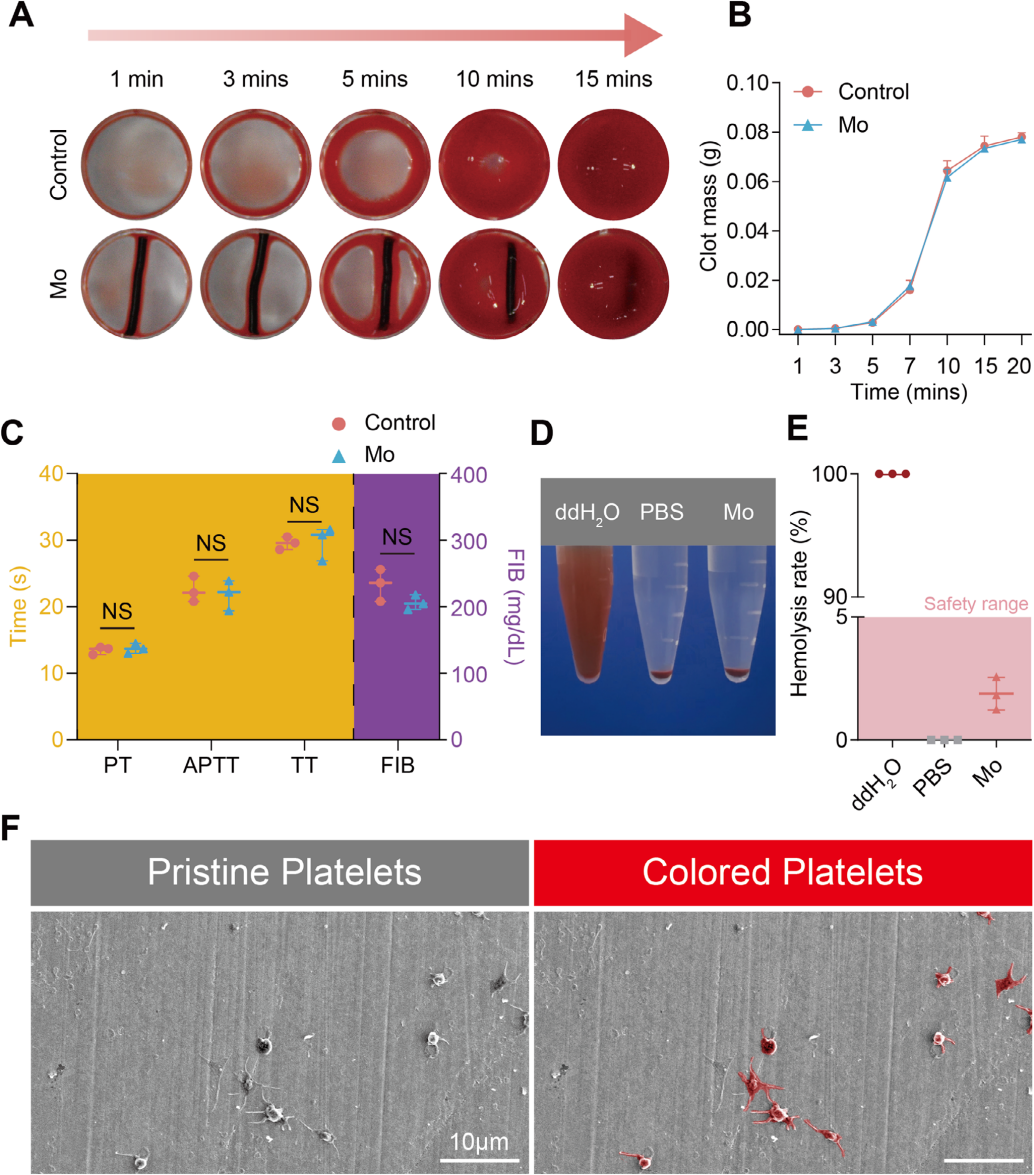
In vitro hemocompatibility of Mo wire. A) Images and B) mass of blood clots after blood mixed with Mo at different time points. C) Coagulation test for activated partial thromboplastin time (APTT), prothrombin time (PT), thrombin time (TT), fibrinogen content (FIB). D) The photos of Mo wire in erythrocyte suspension after centrifuge. E) The hemolysis rate of Mo. F) SEM images of pristine and colored platelets adhesion on Mo.

A well hemocompatibility is of vital importance for endovascular devices. As shown in **Figure** **2**, the A) images and the B) quantitative clot mass analysis reveals that blood coagulation kinetics and thrombus formation in the presence of Mo wire are statistically indistinguishable from the control group. And the coagulation assays (APTT, PT, TT, FIB) in Figure 2C further confirm that Mo exhibits no significant interference with exogenous or endogenous coagulation pathways and fibrinogen functionality. The postcentrifugation images of erythrocyte suspensions (Figure 2D) and hemolysis rate quantification (<5%) (Figure 2E) demonstrate negligible erythrocyte damage, complying with biomedical material safety standards. Moreover, the SEM analysis in Figure 2F shows minimal platelet adhesion on Mo substrate surface, with only sporadic platelets observed and no evidence of extensive activation or pseudopodia formation. These findings collectively affirm the excellent hemocompatibility of Mo wire, highlighting its potential for blood‐contacting medical implants.

### 2.2 In Vitro Cytotoxicity of Mo Wire

**Figure 3:**
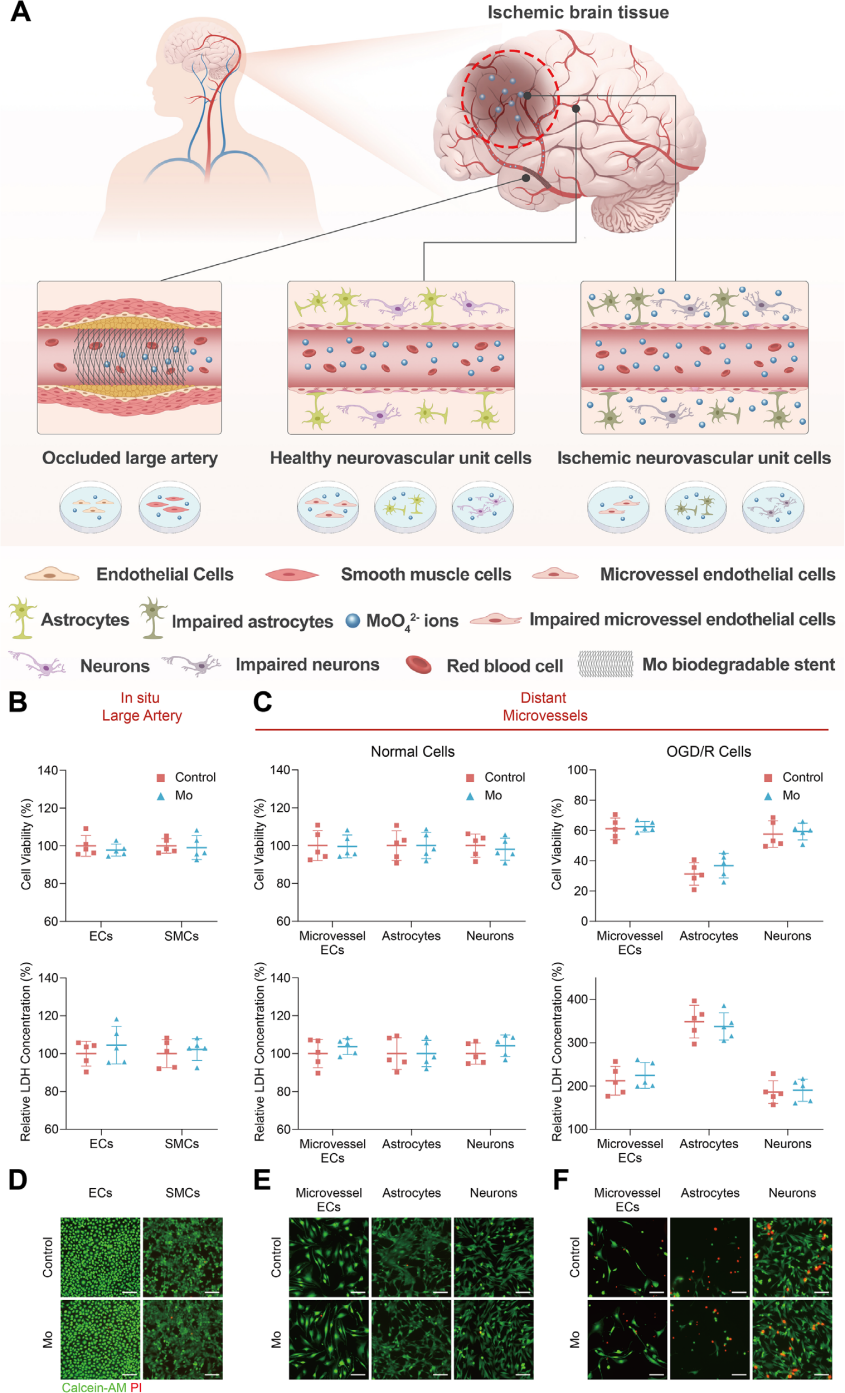
In vitro cytotoxicity of Mo. A) Illustration of the cytotoxicity of Mo degradation production on the large artery (HUVECs, HASMCs) and the on health and ischemic distal NVU (bEnd.3, C8‐D1A, HT22). B) Cell viability and relative LDH concentration of ECs and SMCs, and C) health and OGD/R injured NVU cell (microvessel ECs, astrocytes, and neurons) treated with Mo extracts. The live/dead images for D) ECs and SMCs, and E) health and OGD/R injured NVU cells treated with Mo extracts.

In order to replicate the effects of intravascular Mo implants on the body in clinical patients, we used an in vitro cell model to simulate the in situ effects of implants degradation products on vascular and neurovascular cells. Under stroke conditions with blood–brain barrier (BBB) disruption, both vascular and neural components need to be considered, and the biosafety of Mo should be assessed within the framework of the neurovascular unit (NVU). The experimental design was illustrated in **Figure** **3**A and the utilized cell models include large artery‐associated cells (human umbilical vein endothelial cells, HUVECs; human aortic smooth muscle cells, HASMCs) and neurovascular unit (NVU) cells (microvascular endothelial cells, bEnd.3; astrocytes, C8‐D1A; neurons, HT22) under both healthy and ischemic conditions. The ischemic microenvironment was simulated via oxygen‐glucose deprivation/reperfusion (OGD/R) to mimic cerebral infarction. The CCK‐8 assays and LDH release measurements (Figure 3B,C) demonstrate that Mo extracts caused no significant reduction in cell viability or membrane damage in healthy vascular cells or NVU cells. Notably, while Mo extracts exhibited no cytotoxicity toward OGD/R‐injured NVU cells, they did not confer additional effects against ischemia‐induced damage. The live/dead staining (Figure 3D–F) corroborates these findings, showing minimal cell death in both vascular and NVU cells treated with Mo extracts, regardless of health or ischemic status. Collectively, these results indicate that Mo degradation products are noncytotoxic to vascular and neural cells under physiological and pathological conditions, supporting the safety of Mo implants for intracranial applications, including stenting in large arteries and exposure to ischemic microenvironments.

### 2.3 In Vivo Biosafety of Mo Wire in Healthy Rat Model

**Figure 4:**
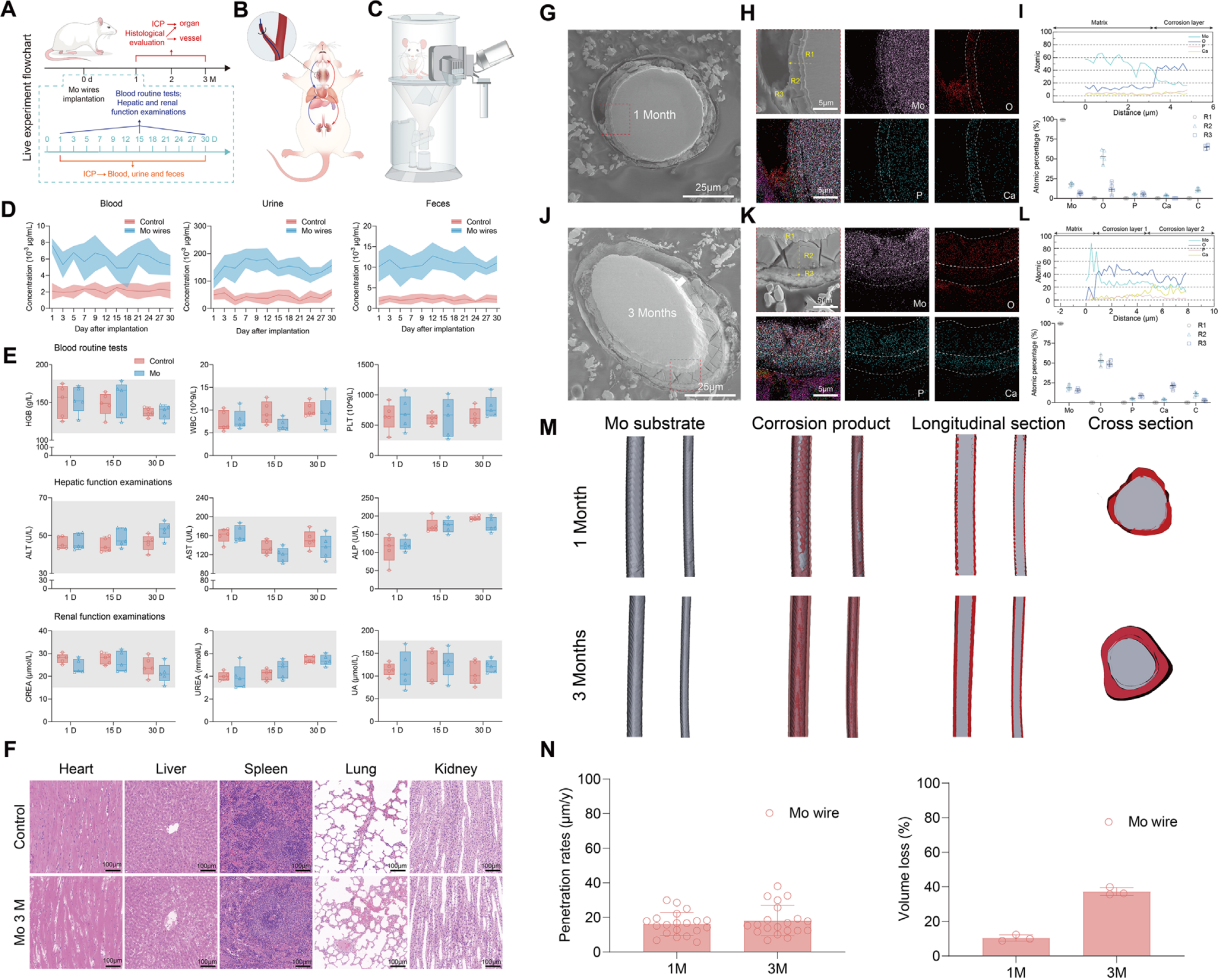
In vivo biosafety of Mo braided stent wire. Illustration of the A) In vivo biosafety study design, B) Mo wire implantation in rat, and C) its urine collection equipment. D) Temporal profiles of Mo ion concentrations in blood, urine, and feces collected at predetermined time points over 1 month. E) Blood routine test and hepatic & renal function. F) H&E‐stained tissue sections (heart, liver, spleen, lung, kidney) from SD rats after 3‐months implantation of Mo wire and SD rats with no implantation as a control. G) Cross‐sectional analysis of explanted Mo wire after 1 month implantation. (Scale bar: 25 µm). H) Elemental mapping (scale bar: 5 µm). I) Line‐scan profile across corrosion layer, and (r1‐r3) Selected‐area EDS point analysis. J) Cross‐sectional analysis of explanted Mo wire after 3 months implantation. (Scale bar: 25 µm). K) Elemental mapping (scale bar: 5 µm). L) Line‐scan profile across corrosion layer, and (r1‐r3) Selected‐area EDS point analysis. M) Cross‐sectional CT reconstruction data of the explanted Mo wire after 1‐ and 3‐months implantation. N) Corrosion rates and volume loss of the explanted Mo wire.

Given the in vitro blood and cerebral cell safety validation of Mo implants, pure Mo wire was inserted into CCA of rats to simulate the state of implanting stents in clinical patients. The specific experimental flowchart is shown in **Figure** **4**A and the braided stent wire implantation was illustrated in Figure 4B. Moreover, blood, urine, and feces from rats were collected continuously within 1 month after Mo wire implantation for ICP testing to determine the in vivo degradation pattern of Mo wire. As demonstrated in Figure 4D, the degradation products of the Mo implant were primarily excreted through the renal system, with urinary elimination representing the dominant clearance pathway. During the degradation of Mo wire, degradation products were metabolized and transferred by passing through the bloodstream to the liver and kidneys. The blood collected at 1, 15, and 30 days after implantation was also used for blood routine and liver and kidney function testing. There was no adverse effects of Mo degradation on blood cell, and hepatic and renal function (Figure 4E). Following 1, 2, and 3 months of Mo wire implantation in the common carotid artery (CCA) of healthy rats, inductively coupled plasma mass spectrometry (ICP‐MS) revealed tissue‐specific accumulation patterns of Mo ions. Related tissues exhibited no statistically significance in Mo content compared to sham controls. And no significant Mo ions accumulation was detected in the spleen, lungs, kidneys, or brain across all timepoints (Figure 4F; and Figures S3 and S4, Supporting Information). The absence of cerebral Mo deposition aligns with the blood–brain barrier's selective permeability, further supporting the neural safety profile of Mo implants.

For cross‐sectional analysis of explanted molybdenum wire (1‐ and 3‐month postimplantation), we employed conductive resin embedding followed by argon ion polishing (However, two technical challenges affected the section quality: 1) Material hardness mismatch between the Mo wire and epoxy resin; 2) Geometric constraints from the small wire diameter. Particularly for the 3‐month samples, these factors caused nonideal sectioning plane. We therefore implemented topographical corrections for corrosion layer thickness measurements (Figure 4G,J). For a cylindrical wire of diameter D = 50 µm (radius R = 25 µm) sectioned at angle θ, the resulting elliptical cross‐section exhibits: Major axis: 2a. Minor axis: 2b = D = 50 µm. Here, we establish a simple mathematical model under the following assumptions:^[^ ^1^ ^]^ the corrosion product layer forms a uniform annular ring around the metal core, and^[^ ^2^ ^]^ the observation direction is parallel to the cross‐sectional normal vector.

The sectioning angle θ is determined from the axial ratio

(1) cosθ=b/a

The corrosion layer forms a concentric elliptical annulus with thickness *t*. At any point P(ϕ) on the ellipse (parameterized by angle ϕ), the apparent thickness d(ϕ) relates to true thickness t through

(2) $$d\;\phi =t*\sqrt{\left(1-{\;\sin}^{2}\theta \cdot \cos^{2}\phi \right)}$$

For two special Case Solutions: (a) Major Axis Endpoint (ϕ = 0°)

(3) $$d_{\mathrm{long}}=t/\cos\theta \;\to t=d_{\mathrm{long}}\;*\cos\theta$$

(b) Minor Axis Endpoint (ϕ = 90°)

(4) $$d_{\mathrm{short}}=t\;\left(\mathrm{direct}\;\mathrm{measurement}\right)$$

Structural and compositional evolution of the corrosion layers was characterized using energy‐dispersive X‐ray spectroscopy (EDS) line‐scans and point analyses. A homogeneous oxide layer dominated by MoO_2_/MoO_3_ formed uniformly across the surface, indicative of controlled anodic dissolution after 1‐month implantation (Figure 4H,I). A stratified corrosion profile emerged for the Mo wire after 3‐month implantation, with an inner MoO_2_/MoO_3_ layer (ongoing anodic dissolution) and an outer calcium‐phosphate deposits (likely from blood‐derived ions) (Figure 4K,L). The measured penetration rates were 16.71 ± 6.67 µm year^−1^ at 1 month and 18.21 ± 8.83 µm year^−1^ at 3 months postimplantation (Figure 4M,N). Statistical analysis confirmed no significant difference between these timepoints, reflecting steady‐state degradation behavior under homeostatic conditions. With respect to the effect of degradation on the surrounding microenvironmental pH, our earlier in vitro studies confirmed that molybdenum corrosion induces only minimal pH fluctuations.^[^ ^11^, ^24^ ^]^ However, we acknowledge that in vivo measurements will still be necessary in future work to provide direct and physiologically relevant evidence.

**Figure 5:**
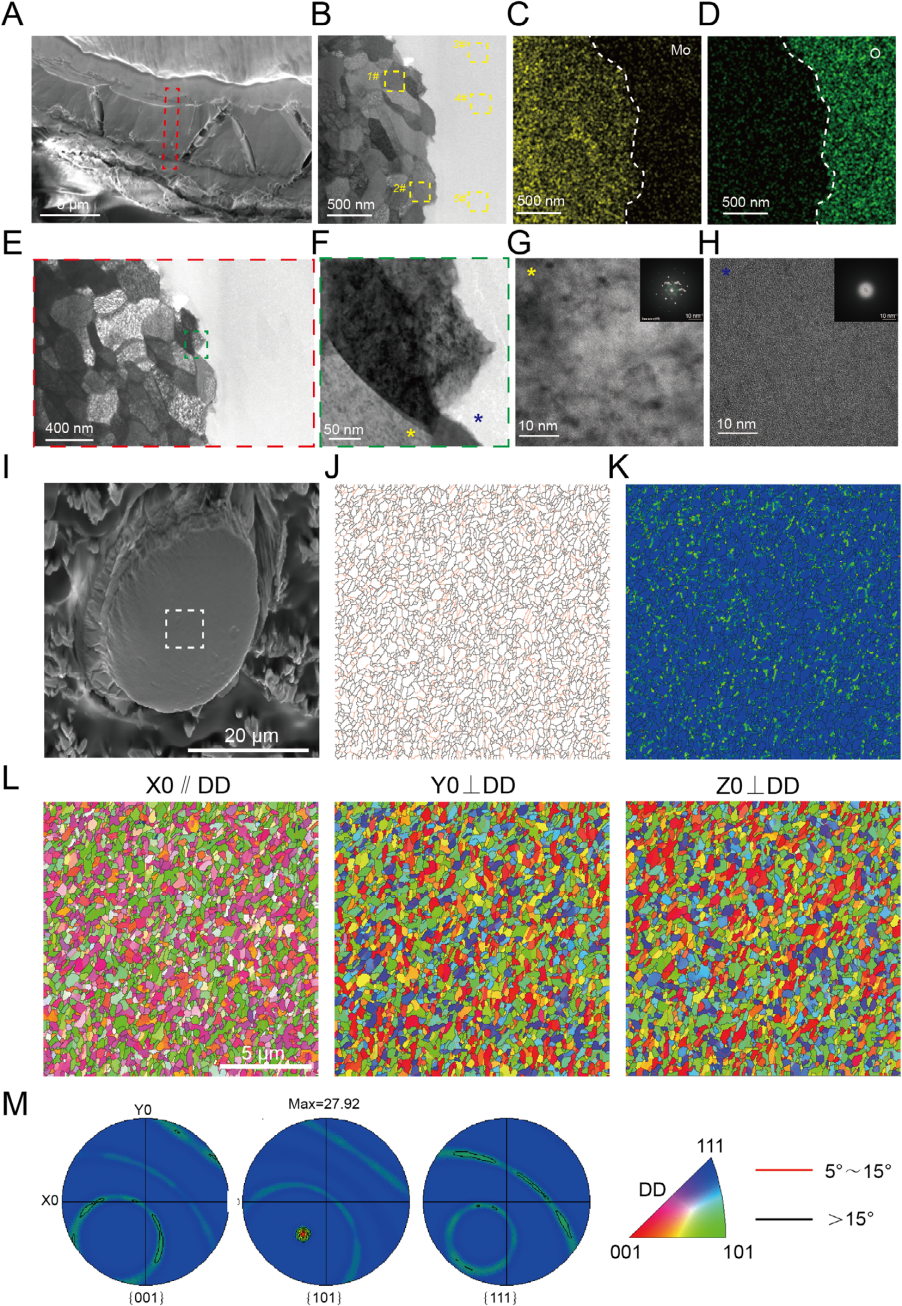
In vivo biodegradation of Mo braided stent wire. A) SEM images of the Focused Ion Beam treated region form TEM analysis of the Mo wire implanted in rat for 3 months. B) TEM image and EDS point analysis regions (the EDS results of each point was shown in Figure S6, Supporting Information). C,D) EDS mapping images, and E,F) bright‐field TEM images at Mo matrix/corrosion product interface. HADDF image and SAED pattern of G) Mo matrix and H) HADDF corrosion products. I) SEM images of the EBSD analysis region (indicated by white box). J) Grain boundary map (black and red lines represent grain boundaries with misorientation angles >15° and ≤15°, respectively). K) Kernel average misorientation (KAM) map. L,M) EBSD pole figure (PF) orientation maps (DD: drawing direction).

To further analyze the interface between the Mo wire matrix and corrosion products, we conducted TEM characterization. The observations revealed uniform corrosion behavior without evidence of transgranular corrosion (**Figure** **5**B,E), demonstrating homogeneous degradation of the metallic matrix. Selected‐area electron diffraction (SAED) patterns in Figure 5G,H confirmed the amorphous nature of corrosion products, which aligns with our previous reports on pure Mo degradation in physiological environments^[^ ^11^ ^]^ and findings from 3D‐printed Mo wire implanted in rabbit abdominal aortae.^[^ ^23^ ^]^ EBSD analysis of the Mo wire after 3‐month implantation in rats revealed the absence of preferred crystallographic orientation texture in the metal matrix (Figure 5L), and the homogeneous distribution of high‐angle grain boundaries (HAGBs, >15°) with elevated grain boundary energy (Figure 5J). This uniform HAGB distribution precludes the formation of preferential corrosion pathways, thereby explaining the observed homogeneous corrosion behavior of molybdenum.

**Figure 6:**
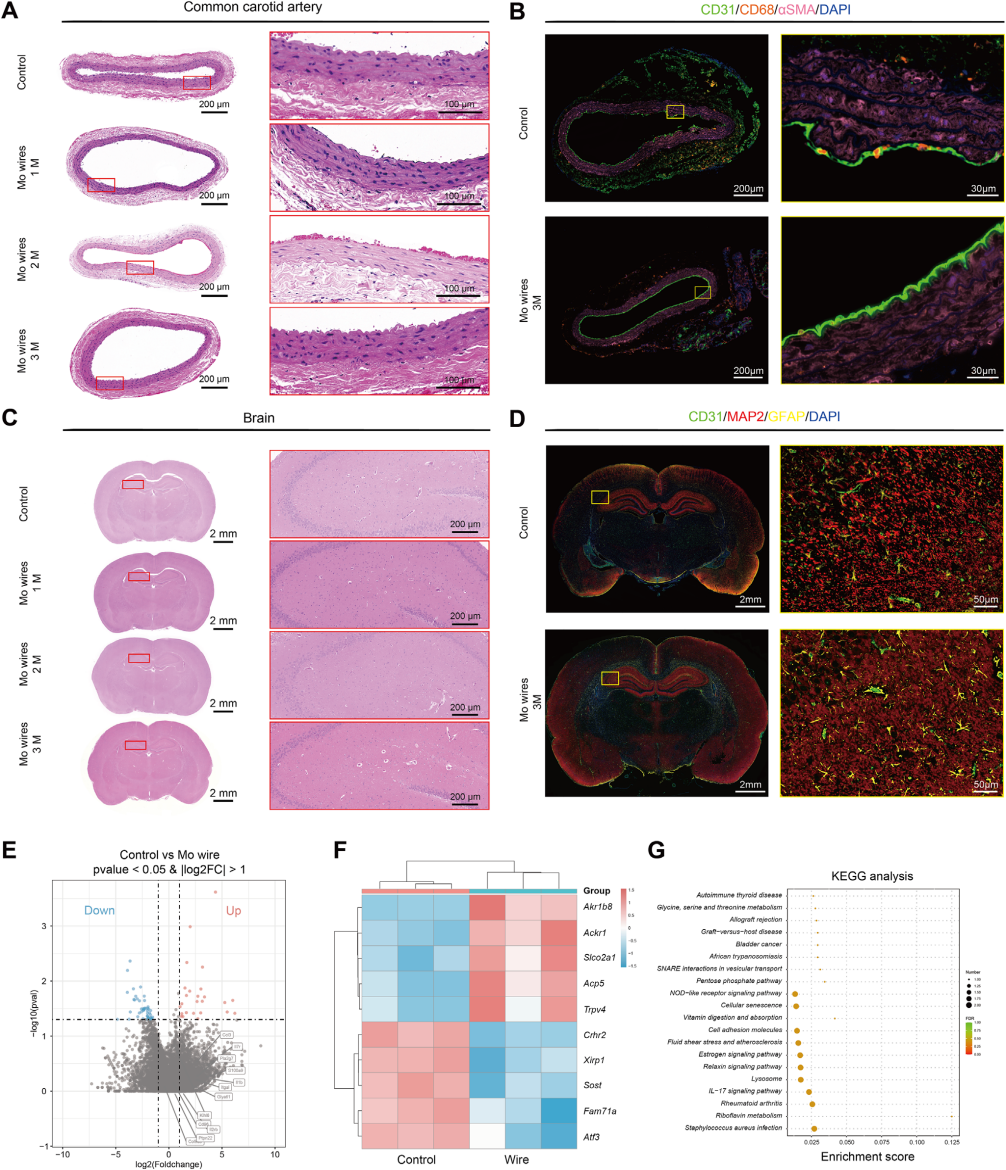
Staining of tissue sections around the implanted Mo braied stent wire. A) H&E‐stained cross‐section of common carotid artery. B) Immunofluorescence staining of common carotid artery section; Markers: CD31 (green), CD68 (orange), αSMA(pink), DAPI (blue). C) H&E‐stained coronal brain section. D) Immunofluorescence staining of coronal brain section, Markers: CD31(green), GFAP (yellow), MAP2 (red), DAPI (blue). E) Volcano plot representation of differential abundance of mRNA expression in in common carotid artery with wire versus Controls. The *x*‐axis indicates the differential expression profiles, plotting the log2(Foldchange). The *y*‐axis indicates ‐log10(*p*‐values) by unpaired two‐tailed *t*‐test. F) Heatmap of differentially expressed mRNA in common carotid artery with wire versus Controls. G) KEGG pathway enrichment analyses. *x*‐axis represents level of statistical significance of enrichment (Fisher exact test). (*n*  =  3).

Furthermore, after Mo wire implantation, no significant morphological changes were observed in the common carotid artery (CCA), brain, or other major organs (**Figure** **6**A,C). Immunofluorescence analysis confirmed that the distribution and morphology of various cell types in both vascular and brain tissues remained physiologically normal, showing no notable differences compared to the control group (Figure 6B,D). It is noteworthy that a small number of CD68‐labeled cells were observed in both the control and experimental vascular tissues. Because CD68 is a macrophage marker, and CD68‐positive cells may also be present under physiological conditions, where they participate in immune surveillance, debris clearance, and maintenance of vascular homeostasis.^[^ ^25^ ^]^ Importantly, no significant inflammatory cell infiltration was detected in vascular tissue samples, indicating that molybdenum wire implantation does not elicit a substantial inflammatory response. Additionally, molybdenum wire implantation did not cause significant effects on surrounding blood vessels (Figure 6E–G). The volcano plot showed almost no significantly differentially expressed genes between the two groups, and the genes that differed between the molybdenum coil group and the control group showed no significant differences when comparing the molybdenum wire group with the control group. KEGG analysis revealed no pathways with high enrichment scores in the molybdenum wire implantation group.

The above results indicated that Mo implants can be safely metabolized in the body, and there were no harmful reactions in the blood, organs, or in situ vessels of the implanted objects.

### 2.4 In Vivo Neurological Safety of Mo Wire in tMCAO Rat Model

**Figure 7:**
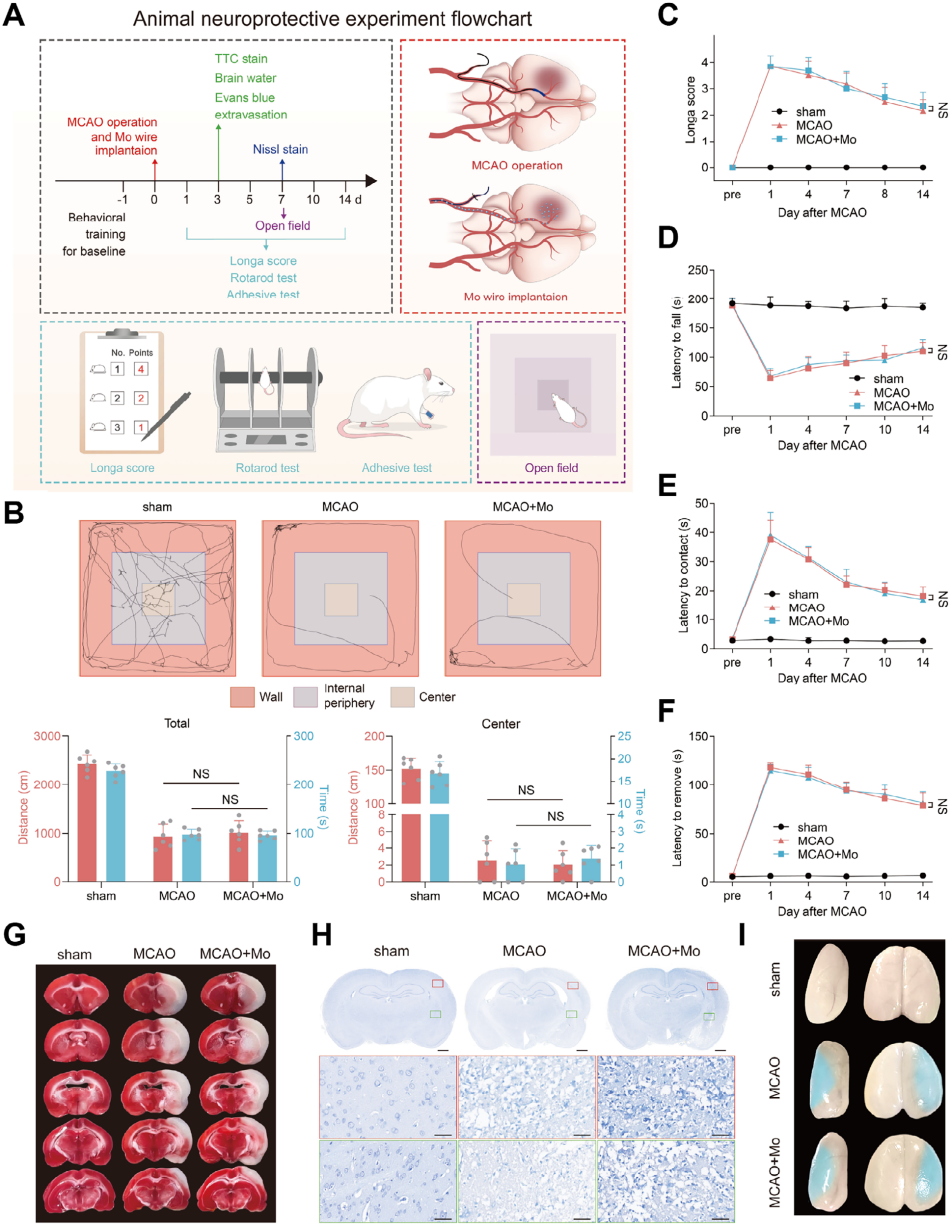
In vivo neurological function effect of Mo braided stent wire. A) The flowchart for in vivo neurological safety of Mo wire in tMCAO rat model. B) Longa score test. C) Rotarod test. D,E) Adhesive contact and removal test. F) Open field test. NS, not significant. G) Brain slices stained with TTC. H) Brain slices with Nissl staining. I) Representative Evans blue extravasation image.

Considering the excellent in vivo safety of Mo wire in health rats, the neurological safety of Mo braided stent wire was further detected in tMCAO rats that simulating AIS patients. The experimental flowchart of this part was shown in **Figure** **7**A. However, according to the comprehensive evaluation of neurological function, Mo wire implantation could not save the damage neurological function (Figure 7B–F; Figure S7, Supporting Information). Meanwhile, Mo wire had no beneficial effect on reducing the volume of cerebral infarction (Figure 7G), saving the integrity of neural cells (Figure 7H), decreasing blood–brain barrier leakage and the degree of cerebral edema (Figure 7I). The quantification analysis results are presented in Figure S8 (Supporting Information). Thus, the possibility of neuro‐safety of Mo implants was confirmed.

### 2.5 In Vivo Effectiveness and Biosafety of Mo Coils in Aneurysm Treatment

**Figure 8:**
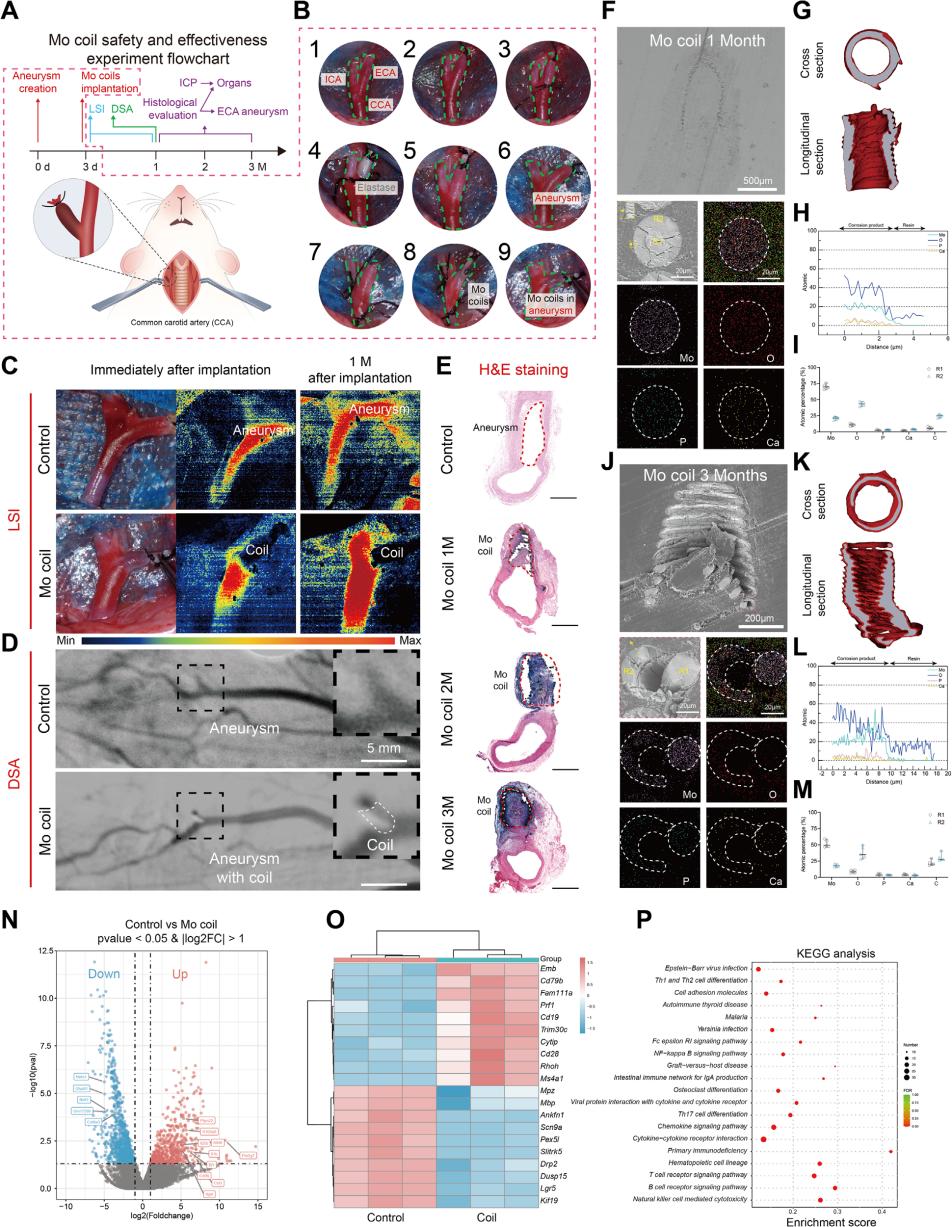
In vivo biosafety of 2D Mo coil. A) The flowchart for in vivo biosafety testing of 2D Mo coil in a rat aneurysm model. B) Creation of aneurysm model in SD rats and implantation of 2D coil. C) Photographs of the aneurysm and laser speckle blood flow distribution before and after coil implantation. D) DSA images pre‐ and post‐2D coil implantation in aneurysm (dashed‐line area clearly delineates coil contour. E) H&E‐stained cross‐sectional slices of the aneurysm (scale bar 500 µm). Cross‐sectional analysis of explanted Mo coil after 1 month implantation. F) Elemental mapping. G) Cross‐sectional CT Reconstruction Data. H) Line‐scan profile across corrosion layer, and I) (R1‐R2) Selected‐area EDS point analysis. Cross‐sectional analysis of explanted Mo coil after 1 month implantation. F) Elemental mapping. G) Cross‐sectional CT Reconstruction Data. H) Line‐scan profile across corrosion layer, and I)(R1‐R2) Selected‐area EDS point analysis. Cross‐sectional analysis of explanted Mo coil after 3 month implantation. J) Elemental mapping. K) Cross‐sectional CT Reconstruction Data. L) Line‐scan profile across corrosion layer, and M) (R1‐R2) Selected‐area EDS point analysis. N) Volcano plot representation of differential abundance of mRNA expression in Coil‐Treated Aneurysms versus Controls. The *x*‐axis indicates the differential expression profiles, plotting the log2(Foldchange). The *y*‐axis indicates ‐log10(*p*‐values) by unpaired two‐tailed *t*‐test. O) Heatmap of differentially expressed mRNA in Coil‐Treated Aneurysms versus Controls. P) KEGG pathway enrichment analyses. *x*‐axis represents level of statistical significance of enrichment (Fisher exact test) (*n*  =  3).

Although Mo wire had no neuroprotective effects, due to the good biocompatibility of Mo implants and their potential as a material for coils to treat cerebral aneurysm, we implanted 2D Mo coils into a modified rat ECA aneurysm model to further investigate in vivo safety and effectiveness of Mo coils. The experimental process is shown in **Figure** **8**A,B. After Mo coil implantation, the blood flow in the aneurysms was immediately blocked, which was verified by LSI (Figure 8C). Moreover, the results of LSI and DSA both indicated that 1 m after implantation, the aneurysms were completely embolized (Figure 8D). The H&E staining of the coils with tissues were presented in Figure 8E. Our results show a progressive increase in neotissue layer thickness over time, with substantial coverage at 1 month and near‐complete uniform neotissue by 2 and 3 months (Figure S12, Supporting Information). Cross‐sectional SEM, EDS, and µCT analysis revealed that the corrosion product layer primarily consisted of a uniform MoO_2_/MoO_3_ composite (Figure 8F–M). After 3 months of implantation, SEM imaging clearly demonstrated mechanical delamination between the remaining metallic wire core and the surrounding corrosion layer (Figure 8J). The measured corrosion rates of the 2D coils were 16.14 ± 6.69 µm year^−1^ at 1 month and 29.73 ± 9.33 µm year^−1^ at 3 months postimplantation (Figure S9, Supporting Information). The accelerated corrosion at the 3‐month timepoint may be attributed to ROS‐mediated degradation triggered by immune responses to the aneurysm‐embolizing coils (Figure 8E). And no significant Mo ions accumulation was detected in the spleen, lungs, kidneys, or brain across all timepoints (Figures S10 and S11, Supporting Information).

Transcriptomic sequencing was performed on aneurysm tissues to elucidate the biological alterations induced by molybdenum (Mo) coil implantation (Figure 8N–P). Comparative analysis revealed 1818 differentially expressed genes (DEGs) in Mo‐treated aneurysms versus controls, comprising 771 upregulated (highlighted in red) and 1047 downregulated genes (blue). Key DEGs associated with device‐typical foreign body reaction (e.g., IL1B, IL2RB, IL7R) and were selectively annotated to avoid visual clutter. The heatmaps of DEGs further demonstrated that Mo coil implantation significantly upregulated CD19, CD28, and other genes involved in cell adhesion. Kyoto Encyclopedia of Genes and Genomes (KEGG) enrichment analysis demonstrated significant activation of pathways including B cell receptor signaling pathway, T cell receptor signaling pathway, and Chemokine signaling pathway, consistent with expected inflammatory responses to implanted devices. This finding is consistent with the tissue responses observed in the H&E‐stained sections of coil‐treated aneurysm tissues in our study (Figure 8E). Gene Ontology (GO) terms enriched in biological processes included leukocyte activation and immune response.

Notably, comparative transcriptomic profiling of parent vessel tissues adjacent to Mo wire showed no significant DEGs versus controls (FDR > 0.1), indicating localized biological effects restricted to the aneurysmal sac. These findings suggest that Mo coils elicit a transient, device‐typical foreign body reaction within aneurysms while preserving the integrity of surrounding vasculature, which is a critical safety feature for neurovascular applications.

## 3 Discussion

This study systematically investigates the neural biocompatibility and feasibility of biodegradable Mo endovascular implants, specifically intracranial stents and aneurysm coils, using tMCAO and aneurysmal rat models. Comprehensive hemocompatibility assessments confirmed the inert interaction of Mo with blood components, while in vitro cytotoxicity assays demonstrated no adverse effects of Mo extracts on large‐vessel cells (HUVECs, HASMCs) or NVU cells (bEnd.3, C8‐D1A, HT22) under both healthy and ischemic conditions. In vivo evaluations revealed no systemic toxicity, as evidenced by unchanged blood biochemistry, hepatic/renal function, and histopathology of major organs (including the brain) following Mo wire implantation into the rat CCA. Critically, Mo braided stent wire implantation did not exacerbate neurological deficits in cerebral ischemic rats. Notably, Mo coils exhibited effective aneurysm embolization in rat models without triggering inflammatory or thrombotic complications. These findings collectively position Mo as a promising candidate for neurovascular interventions, offering dual functionality as a mechanically stable and biologically compatible material.

### 3.1 Potential Pros and Cons of Mo for Making Biodegradable Neurovascular Implants

While magnesium (Mg), iron (Fe), and zinc (Zn) alloys have been explored as biodegradable materials for neurovascular implants, their limitations hinder clinical translation. Mg alloys suffer from rapid degradation rates, generating hydrogen gas and local pH shifts that risk neural tissue damage. Fe alloys exhibit slow corrosion, potentially leading to prolonged inflammatory responses, while Zn alloys often lack sufficient mechanical strength for intracranial applications. In contrast, molybdenum (Mo) offers a balanced degradation profile, combining moderate corrosion rates with mechanical stability, making it particularly suitable for the dynamic cerebrovascular environment. Its inherent radiopacity further facilitates intraoperative imaging, which is a critical advantage over radiolucent Mg/Zn alloys. However, challenges persist in processing Mo into intricate neurovascular devices due to its high melting point, which complicates traditional methods like laser‐cutting thin‐walled tubes. To address this, our study leverages advanced wire‐braiding techniques to fabricate Mo‐based stents and coils, bypassing conventional machining limitations. Despite these advancements, current biocompatibility evaluations of Mo remain largely confined to healthy animal models, focusing on vascular corrosion and hemocompatibility, with limited data on its interaction with neural cells or safety in disease contexts (e.g., ischemic or aneurysmal conditions). Our work bridges this gap by systematically assessing Mo's neural biocompatibility in both physiological and pathological settings, demonstrating its safety in tMCAO and aneurysm models. Nevertheless, further studies are warranted to optimize Mo's processing scalability and evaluate long‐term neuroinflammatory responses in large‐animal models.

### 3.2 Hemocompatibility and Vascular Cell Compatibility of Mo Degradation Products

The localized degradation of Mo implants within blood vessels necessitates rigorous evaluation of their interactions with blood components and vascular cells. Our in vitro hemocompatibility assessments demonstrated that Mo implants exhibit excellent biocompatibility, causing negligible hemolysis (<5%), and minimal platelet adhesion/activation, consistent with prior findings from 3D‐printed Mo powder stents.^[^ ^23^ ^]^ Notably, this study provides the first evidence that Mo degradation products do not disrupt coagulation pathways, as evidenced by unaltered activated partial thromboplastin time (APTT), prothrombin time (PT), thrombin time (TT), and fibrinogen (FIB) levels compared to controls.

While Redlich et al. reported endothelial cell (EC) dysfunction at extremely high MoO_3_ concentrations (2.5 m,^[^ ^26^ ^])^ our experiments using physiologically relevant Mo extract doses that derived from in vitro degradation confirmed no cytotoxicity toward ECs or SMCs. Critically, the 2.5 m MoO_3_ concentration employed by Redlich et al. exceeds the calculated in vivo Mo ion release by over three orders of magnitude,^[^ ^26^ ^]^ underscoring that their findings do not contradict the safety profile observed here at clinically pertinent doses.

In vivo implantation of Mo wire further validated their vascular compatibility, with histopathological analysis revealing preserved vessel structure at implantation sites. These results align with Bian et al.’s report on Mo stent biocompatibility^[^ ^23^ ^]^ and partially corroborate Sikora‐Jasinska et al.’s observation of comparable endothelialization between Mo and control groups.^[^ ^17^ ^]^ The minor discrepancy in endothelialization metrics reported by Sikora‐Jasinska et al. may stem from technical artifacts during frozen section preparation rather than intrinsic material properties,^[^ ^17^ ^]^ emphasizing the need for standardized histological methodologies in biodegradable metal evaluations.

### 3.3 The Safety of Mo Degradation Products on Brain and Other Major Organs

Current research on biodegradable Mo implants predominantly focuses on cardiovascular and peripheral vascular applications, leaving a critical knowledge gap in cerebrovascular contexts. Given the unique sensitivity of the brain microenvironment, evaluating the neurobiological impact of Mo degradation products is essential for intracranial device translation. This study pioneers the assessment of Mo's effects on neurovascular unit (NVU) cells in vitro, demonstrating that Mo extracts at physiological concentrations elicit no cytotoxicity in microvascular endothelial cells (bEnd.3), astrocytes (C8‐D1A), or neurons (HT22) under normal or ischemic‐mimetic conditions. To simulate clinical scenarios, Mo wire was implanted into the rat common carotid artery (CCA), enabling degradation products to enter cerebral circulation directly. Histopathological analysis confirmed no adverse morphological alterations in brain tissue, affirming the localized neurocompatibility of Mo.

Systemically, Mo degradation products enter circulation and interact with major organs. Hematological profiling over 30 days postimplantation revealed no deviations in erythrocyte, leukocyte, or platelet counts, consistent with Schauer et al.’s findings in peripheral Mo implants.^[^ ^27^ ^]^ While Sikora‐Jasinska et al. reported qualitative glomerular structural changes in rat kidneys,^[^ ^17^ ^]^ our histopathological and functional analyses detected no significant renal or hepatic abnormalities, aligning with Schauer et al.’s safety data.^[^ ^27^ ^]^ This discrepancy may arise from differences in Mo degradation kinetics, implant geometry, or interspecies metabolic variability. Notably, as renal excretion is the primary route for Mo clearance, extended studies are warranted to evaluate long‐term renal tolerance. Additionally, Mo implantation induced no pathological changes in the heart, lungs, or spleen, further supporting its systemic biocompatibility.

### 3.4 Metabolic Analysis

Understanding the biodistribution and clearance pathways of emerging biomaterials represents a fundamental challenge in developing next‐generation bioresorbable implants. Despite molybdenum's growing promise as a biodegradable metallic material, the long‐term in vivo fate of Mo‐based implants remains unexplored—a critical knowledge gap impeding clinical translation. Giussani's biokinetic modeling demonstrated molybdenum (Mo) preferentially accumulates in liver and kidneys through albumin‐bound transport, with efficient renal clearance preventing systemic overload. Novotny and Turnlund (2007) quantified this homeostasis in humans, showing ≈60%–70% of absorbed Mo is excreted renally within 24 h, while residual Mo redistributes to metabolic pools (liver, bones) without exceeding physiological thresholds. Together these studies establish Mo's inherent metabolic compatibility, and its rapid renal clearance maintains systemic balance even during implant degradation.

### 3.5 The Influence of Mo Degradation Products on Ischemic Brain Tissue

Acute ischemic stroke (AIS) patients often require stent‐assisted recanalization post‐thrombectomy to restore cerebral perfusion, necessitating evaluation of Mo's effects in ischemic environments. To bridge this translational gap, we implanted Mo wire in a tMCAO rat, mimicking stent placement in AIS patients.

Notably, while Mg implants have demonstrated neuroprotective benefits via Mg^2+^ and H_2_ release during degradation, Mo's biological role is more nuanced. As a trace element, Mo serves as a cofactor for critical metabolic enzymes, including xanthine oxidoreductase (XOR) and aldehyde oxidase (AOX), which regulate redox homeostasis. XOR catalyzes uric acid (UA) production, a potent antioxidant implicated in neuroprotection. Preclinical studies highlight UA's therapeutic potential in AIS,^[^ ^28^ ^]^ and clinical data correlate elevated UA levels with improved stroke outcomes.^[^ ^29^ ^]^ Intriguingly, prior reports suggest a link between serum Mo and UA concentrations, raising the hypothesis that Mo implant degradation could enhance UA synthesis and mitigate ischemic injury.

Contrary to this hypothesis, our study observed no significant increase in serum UA levels or neurofunctional improvement in tMCAO rats within 1 month of Mo implantation. These findings suggest that short‐term Mo degradation may not sufficiently modulate XOR activity or UA production to confer neuroprotection. However, the enzymatic role of Mo in XOR/AOX also poses a dual‐edged sword: excessive Mo release could theoretically amplify oxidative stress by promoting reactive oxygen species (ROS) generation. Thus, optimizing Mo's degradation kinetics to balance enzymatic regulation and oxidative risk remains critical.

Despite the absence of acute neuroprotection, this work underscores the need for long‐term studies exploring Mo's enzymatic interactions in chronic ischemia and its potential synergy with adjuvant therapies. Future investigations should also delineate dose‐dependent effects of Mo ions on XOR/AOX activity and validate these mechanisms in large‐animal models with clinically relevant degradation rates.

### 3.6 Mo Coils as the Potential Materials for Aneurysm Treatment

Hemorrhagic stroke, predominantly caused by ruptured intracranial aneurysms, poses a significant global health burden. Endovascular coil embolization remains the gold standard for aneurysm treatment, yet permanent metallic coils (e.g., platinum) carry risks of chronic inflammation, recurrence, and imaging artifacts. Biodegradable coils, designed to transiently occlude aneurysms while promoting endothelialization, have emerged as a promising alternative. However, existing biodegradable materials, such as magnesium alloys, often exhibit rapid degradation or insufficient mechanical stability. Leveraging the verified biocompatibility of molybdenum (Mo) wire, this study pioneers the fabrication and evaluation of braided Mo coils in a rat aneurysm model to assess their embolic efficacy and biological safety.

### 3.7 Corrosion Behavior of Mo Braided Stent Wire

The in vivo corrosion rate of Mo significantly exceeds in vitro measurements, with both volume loss and penetration rate demonstrating comparable magnitudes to zinc alloys cardiovascular stents,^[^ ^30^ ^]^ which is a noteworthy contrast to conventional biodegradable metal behavior. Typically, in orthopedic studies of biodegradable implants, the degradation rate in vivo tends to be slower than in vitro conditions.^[^ ^31^ ^]^ The faster in vivo corrosion of molybdenum implants (vs in vitro) may involve several plausible mechanisms requiring further investigation: i) Neutrophil‐derived ROS accelerate molybdenum corrosion, as demonstrated in our previous work,^[^ ^11^ ^]^ ii) pulsatile shear stress promoting corrosion product layer detachment,^[^ ^32^ ^]^ and iii) Protein adsorption may form localized galvanic cells, potentially accelerating corrosion of the metal substrate.^[^ ^33^ ^]^ While these preliminary observations highlight molybdenum's unique potential as a biodegradable neurovascular material—particularly its excellent radiopacity and uniform degradation pattern—future studies must systematically validate each mechanism through controlled in vivo models (e.g., ROS‐inhibited animals, flow‐mimicking bioreactors) before clinical translation can be considered.

### 3.8 Future Directions for Mo Wire Braided Neurovascular Devices

This study provides foundational evidence for the neural biocompatibility and feasibility of Mo metal in cerebrovascular applications. To advance translational progress, the following critical research avenues warrant prioritization:

#### 3.8.1 Development of Functional Mo Stents for Atherosclerotic Models

While Mo wire serves as simplified surrogates for stent evaluation, clinically relevant devices must replicate the mechanical and hemodynamic properties of vascular stents. Future work should focus on fabricating laser‐cut or braided Mo stents with optimized strut thickness and radial strength to address atherosclerotic stenosis. Implantation into rabbit iliac or carotid artery atherosclerosis models (e.g., high‐fat diet + balloon injury) will enable assessment of Mo's interaction with plaque microenvironments, including endothelialization rates, neointimal hyperplasia, and long‐term patency. Concurrently, computational fluid dynamics (CFD) simulations could predict stent‐induced flow alterations in tortuous cerebrovascular anatomies.

#### 3.8.2 High‐Fidelity Neurovascular Safety Validation

Although Mo implants showed no acute neurotoxicity in rodent models, their chronic effects on the human brain, particularly in ischemic or aging contexts, require evaluation in higher‐order species. Implanting degradable Mo stents into cerebral arteries of nonhuman primate (NHP) models of AIS^[^ ^34^ ^]^ (e.g., endovascular occlusion‐reperfusion) would provide critical insights into neuroinflammatory responses, blood–brain barrier integrity, and synaptic plasticity using advanced techniques like PET‐MRI and transcriptomic profiling.

#### 3.8.3 Optimization of 3D Mo Coils for Complex Aneurysm Morphologies

Current 2D Mo coils, while effective in rodent models, lack the volumetric packing density required for human‐sized aneurysms. Utilizing braiding or shape‐memory techniques to fabricate 3D Mo coils (e.g., helical, spherical configurations) and testing them in rabbit elastase‐induced carotid aneurysm models^[^ ^35^ ^]^ will better mimic clinical challenges. Real‐time imaging (e.g., DynaCT) can monitor coil compaction and degradation, while histology (Masson's trichrome, α‐SMA staining) will assess fibrous tissue integration and parent vessel remodeling.

#### 3.8.4 Expanding Mo's Role in Advanced Neurointerventional Devices

Beyond coils, Mo's mechanical robustness and radiopacity make it ideal for next‐generation flow diverters^[^ ^36^ ^]^ and intrasaccular disruptors.^[^ ^37^ ^]^ For instance, braided Mo flow diverters could combine transient flow redirection with biodegradability, addressing the chronic foreign body risks of permanent polymer devices.^[^ ^38^ ^]^ Hybrid designs, such as drug‐eluting Mo surfaces loaded with antiproliferative agents (e.g., sirolimus), may further inhibit endothelial hyperplasia while leveraging Mo's corrosion‐controlled release kinetics.

#### 3.8.5 Alloy Design to Enhance Processability and Functionality

Current Mo biomedical applications are limited to pure metal forms due to processing challenges. Strategic alloying with rhenium (Re, ≈3–5 wt%) or zirconium (Zr, ≈0.5–1 wt%) could improve ductility and reduce recrystallization temperatures, enabling laser‐cutting of thin‐walled stent tubes. Additionally, trace titanium (Ti) additions may enhance endothelial cell adhesion via surface oxide modulation. However, rigorous biocompatibility screening is essential to avoid introducing cytotoxic alloying elements (e.g., nickel).^[^ ^39^, ^40^ ^]^

### 3.9 Conclusions

This study establishes Mo metal as a neurobiocompatible and viable candidate for biodegradable neurovascular implants, addressing critical gaps in stroke intervention. Comprehensive in vitro and in vivo evaluations confirmed Mo metal's excellent hemocompatibility, with negligible hemolysis (<5%), minimal platelet activation, and preserved coagulation profiles. Cytotoxicity assays demonstrated no adverse effects on vascular or neurovascular unit (NVU) cells, even under ischemic conditions. Systemic safety assessments in rodent models revealed no histopathological abnormalities in major organs, with Mo ions accumulation restricted to the liver and heart (likely mediated by physiological metabolic pathway) and absent in brain tissue, underscoring its neural inertness. Notably, Mo 2D coils achieved stable aneurysm occlusion in rats, accompanied by progressive endothelialization and transient inflammation, while Mo braided stent wire exhibited no exacerbation of neurological deficits in ischemic models. Unlike Mg/Fe alloys, Mo metal's balanced degradation kinetics and inherent radiopacity circumvent risks of rapid gas generation or imaging artifacts. Future work should prioritize large‐animal validations, long‐term degradation monitoring, and clinical‐grade device optimization. Collectively, these findings position Mo metal as a transformative material for next‐generation neurovascular devices, harmonizing biodegradability, and safety in stroke management.

## 4 Experimental Section

### Materials and Devices

Commercial pure Mo (≥99.95 wt%) ultrafine wire with a diameter of 50 µm was purchased from Jinduicheng Molybdenum Co., Ltd., China. The Mo braided stent prototype is woven using a braiding machine with controlled tension to achieve the desired mesh density. The braided structure is then shape‐set on a mandrel and heat‐treated at ≈1100–1400 °C under vacuum to stabilize its geometry. The 2D Mo coil prototype is prepared by wind Mo wire onto a precision mandrel using a computer‐controlled coiling machine to form helical springs with a defined pitch and outer diameter (**Table** **1**). The coiled structure undergoes shape‐setting heat treatment at ≈1100–1300 °C under vacuum to stabilize its geometry (Figure S13, Supporting Information).

**Table 1:** Analyzed compositions of experimental pure Mo.

Mo	C	P	Fe	Al	Si	Ca	Mg	Ni	O	N
≥99.95	≤0.0009	≤0.0004	≤0.0013	≤0.002	≤0.002	≤0.0006	≤0.0003	≤0.0007	≤0.004	≤0.001

### In Vitro Hemocompatibility Evaluation–Coagulation Test

First, thrombus weight and clotting time were measured according to a pre‐existing methodology.^[^ ^41^, ^42^ ^]^ Briefly, to reactive uncoagulated citrated blood, 10% v/v 0.1 m CaCl_2_ was added to blood. Each Mo wire (length 3.0 ± 0.2 mm) received 40 µL of activated blood, which was then left to respond for 1, 3, 5, 7, 10, 15, or 20 min. 120 µL of a 0.109 m sodium citrate solution was added at each time point to stop clotting. The clotted blood was isolated by removing remaining liquid. Then, the clotted blood in each test well was photographed and weighed.

Then, the activated partial thromboplastin time (APTT), prothrombin time (PT), thromboplastin time (TT), and fibrinogen content (FIB) of blood treated with Mo were measured based on a previous method.^[^ ^43^ ^]^ In short, an anticoagulant vacuum tube was used to collect fresh blood from rats, and platelet‐poor plasma (PPP) was obtained by centrifuging the fresh blood. Then Mo coils were added into 1 mL PPP. After 30 min of incubation at 37 °C, the PPP was moved to the sample tube for usage. Finally, APTT, TT, PT, and FIB were measured using an automatic coagulometer.

### Hemolysis Rate Assay

A published strategy was used to calculate the hemolysis ratio.^[^ ^44^ ^]^ The erythrocytes were suspended by slowly blowing with a dropper after the fresh anticoagulant rat blood (1 mL) was gradually added to PBS (10 mL). The erythrocytes were obtained by centrifugation. The precipitated erythrocytes were diluted to create a 4% v/v suspension in PBS. The Mo wire was added to erythrocyte suspension (500 µL), and the combination was incubated for 3 h at 37 °C. After centrifuging the suspension, the supernatant was collected. The absorbance of the supernatant was measured at a wavelength of 540 nm. The erythrocyte suspensions in the positive and negative control groups were incubated with ddH_2_O and PBS, respectively. The following formula was used to calculate the hemolysis ratio: (the optical density of Mo implant group—the optical density of negative control group)/(the optical density of positive control group—the optical density of negative control group) × 100%.

### Platelet Adhesion Test

Platelet adhesion test was conducted according to a previous method.^[^ ^43^ ^]^ Platelet‐rich plasma (PRP) was obtained by centrifuging fresh blood from rats. After that, Mo wires were incubated for 2 h at 37 °C in 500 µL of PRP. After the PRP was removed and the Mo wires were washed three times with phosphate‐buffered saline (PBS), they were fixed with 4% glutaraldehyde in PBS for 24 h at 4 °C. Following a PBS solution wash, the Mo wires were dried and dehydrated using a series of gradient physiological alcohol/PBS solutions (25, 50, 75, 85, 95, and 100 V/V%). Last, scanning electron microscopy (SEM) was used to analyze the adhesion of platelets on the surface of Mo wire.

### In Vitro Cytocompatibility Experiments—Large Artery Related Cells

Large artery mainly includes endothelial cells (ECs) and smooth muscle cells (SMCs). The cytotoxicity effects of pure Mo on these cells were evaluated using human umbilical vein ECs (HUVECs) and human artery SMCs (HASMCs). Briefly, Mo extracts for culture were prepared by immersing pure Mo sheets into 10% fetal bovine serum (FBS) and 1% penicillin/streptomycin supplemented Dulbecco's modified Eagle's medium (DMEM) under standard cell culture conditions (95% relative humidity, 37 °C and 5% CO_2_) for 24 h. Cells seeded in 96‐well plates were cultured with Mo extracts for 24 h. Cells cultured with normal DMEM with FBS and antibiotics were set as control. The cytotoxicity was evaluated using the cell count kit‐8 (CCK‐8) assay, lactic dehydrogenase (LDH) release assay and Live/Dead Cells Staining assay (assay kit provided by Beyotime, China). For CCK‐8 assay, 10 µL of CCK‐8 reagent was applied to each test well for 2 h. The optical density (OD) of each well was determined at 450 nm by a microplate reader (ThermoFisher Scientific, USA). LDH release assay was conducted by incubating the cell supernatants with the assay kit solution following measuring color change at 490 nm through spectrophotometer (ThermoFisher Scientiffc, USA). For Live/Dead Cells Staining assay, each cell well received a mixture of PI and Calcein‐AM solution, which was then incubated for 0.5 h. The cells were examined using a fluorescent microscope (Nikon, Japan). Living cells were stained green with Calcein‐AM, and dead cells were labeled red with PI.

### Neurovascular Unit Cells

The NVU cells evaluated in this study included microvessel ECs (bEnd.3), astrocytes (C8‐D1A), and neurons (HT22). Each kind of cell undergone normal and oxygen glucose deprivation/reperfusion (OGD/R) injury treatment. Normal treated cells were cultured under standard condition to preset convergence level in 96‐well plate. OGD/R treatment was used to simulate ischemia‐reperfusion injury.^[^ ^45^ ^]^ Briefly, the confluent cells' growth medium was switched to glucose‐free DMEM, and they were then placed in an anaerobic chamber with 5% CO_2_ and 95% N_2_ at 37 °C for 4 h. The cells were then placed back in a normoxic condition for 24‐h reoxygenation at 5% CO_2_/95% air after the media was changed to standard DMEM containing glucose. Both normal and OGD/R treated cells were incubated with Mo extracts for 24 h. Last, cells were test with CCK‐8 assay, LDH release assay, and Live/Dead Cells Staining assay as described above.

### Animal Experiments

Adult male Sprague‐Dawley (SD) rats weighing 300 ± 20 g were used in the present study. All experimental procedures were authorized by the Capital Medical University's Institutional Animal Investigation Committee, which made sure that all animal treatments strictly followed the National Institutes of Health's guidelines for the Care and Use of Laboratory Animals.

### Mo Wire Implantation in Healthy Rat for Biosafety Analysis–Surgery Process and Group Setting

The implantation procedure of metal wire was referred to the previous study.^[^ ^20^ ^]^ Following a 7‐day period of adaption and overnight fasting, rats were anesthetized with 5% enflurane and maintained with 2±1% enflurane. Then, the rats were placed in a supine posture, and a shaver was used to remove the hair from their neck. Cotton balls soaked in iodophor were used to disinfect the neck. The right CCA, internal carotid artery (ICA), and ECA were then separated with micro forceps after the tiny, ≈1‐cm incision was made at the midline of the necks with surgical scissors. The microscissor was used to sever the ECA and presanitized 1 cm Mo wire was inserted into CCA in reverse through the ECA opening. Last, Mo wire was fixed onto the ECA to ensure long‐term exposure to the blood flow environment of CCA. The Mo wire implanted rats were set as Mo wire group. The rats without Mo wire implantation were set as control group.

### In Vivo Mo Wire Degradation Analysis

The in vivo Mo elemental contents in blood, urine, feces, and organs were measured based on the method proposed by Matusiewicz et. al.^[^ ^46^ ^]^ Blood was collected at predetermined time points for blood routine test and hepatic and renal function tests. Rats were manually restrained, and after facial hair removal and disinfection, a polymer needle was used to puncture and collect blood from the targeted facial veins. For urine and feces, each rat was individually housed in a separate metabolic cage, and their naturally excreted urine and feces were collected at pre‐set time points. For organs, rats were sacrificed, and their organs (brain, heart, liver, spleen, lung, and kidney) were extracted. Then, the feces and a portion of the each collected organs were cut off for weighing, and were placed into a digestion bottle made of Teflon. Next, after adding HNO_3_ and H_2_O_2_, the blood, urine, feces, and the portion of organs was digested using a microwave system. Finally, the concentrations of Mo were measured using inductively coupled plasma optical emission spectroscopy (iCAP6300, ThermoFisher, USA).

Mo wires were retrieved after 30, 60, and 90 days of implantation, with five wires collected at each time point. They were pressure‐perfused with saline to remove blood and then immersed in 2.5% glutaraldehyde for 12 h. Subsequently, dehydration was carried out using graded ethanol (20%, 40%, 60%, 80%, 95%, 100%; 30 min for each grade).

To characterize the biodegradation of the wire, the explanted wires were analyzed by computed tomography, using a µCT 45 desktop microCT scanner (SCANCO MEDICAL, Switzerland). The scanning parameters for the computed tomography scans were voltage 70 kV, current 150 µA, voxel size 6 µm, rotation step 0.2 °. 3D reconstruction of the Mg wire was conducted, and the volumes of the corrosion layer and remaining magnesium matrix were calculated using Mimics software (Mimics 10.01, Materialise Mimics). The remaining volume of the molybdenum matrix was calculated using the following formula

(5) $$V_{R}=V_{\mathrm{Mo}}/\left(V_{\mathrm{Mo}}+V_{\mathrm{corrosion}}\right)$$

where *V*_R_ is the volume proportion of the uncorroded part of the molybdenum wire after being extracted. *V*_Mo_ is the volume proportion of the uncorroded part of the molybdenum wire after being extracted. *V*_corrosion_ is the volume proportion of the corrosion layer volume of the molybdenum wire after being extracted.

The extracted molybdenum wire was embedded in conductive resin. The cross‐section perpendicular to the drawing direction of the tubes was polished using Ar+ ions at 1 keV for 30 min in an ion milling system (Leica EM RES102). Grain boundary maps and inverse pole figures were obtained through electron backscatter diffraction (EBSD) analysis using an EDAX Velocity Super system. For SEM observation (Hitachi S4800, Japan), the wire cross‐sections were sputter‐coated with gold. The chemical composition of the corrosion product layer was analyzed using the attached energy‐dispersive X‐ray spectroscopy (EDS) detector.

Furthermore, Polished samples were further ion‐beam milled using Helios 5 Dual Beam with 10 KeV. Samples were plasma cleaned before visualized under a high‐resolution high‐angle annular dark‐field mode (HAADF) at 300 kV using a scanning transmission electron microscopy (FEI Titan G2 60‐300 ChemiSTEM).

### Blood Routine Tests

Blood was collected as mentioned above for blood routine tests using automatic hematology analyzer (BC‐10, Mindray, China) including the concentration of red blood cells (RBC), hemoglobin (HGB), white blood cells (WBC), and platelets (PLT). For RBC and HGB, hematocrit (HCT), mean corpuscular volume (MCV), mean corpuscular hemoglobin (MCH), mean corpuscular hemoglobin concentration (MCHC), red cell distribution width coefficient of variance (RDW‐CV), and red cell distribution width standard deviation (RDW‐SD) were also tested. For WBC, the concentration and proportion of neutrophils (Neu#, Neu%), lymphocyte (Lym#, Lym%), monocyte (Mon#, Mon%), eosinophils (Eos#, Eos%), and basophils (Bas#, Bas%) were further measured. For PLT, mean platelet volume (MPV), platelet distribution width (PDW), and plateletcrit (PCT) was simultaneously detected.

### Hepatic and Renal Function Examination

Rat blood was collected for liver and kidney function testing through an automated biochemical analyzer (BS‐280, Mindray, China). Specifically, liver function indicators included alanine aminotransferase (ALT), aspartate aminotransferase (AST), and alkaline phosphatase (ALP), and kidney function indicators composed of creatinine (CREA), uric acid (UA), urea (UREA), and γ‐glutamyl transpeptidase (γ‐GT).

### Histological Evaluation of Organs and Vessels

Following euthanasia, the brain, heart, liver, spleen, lungs, kidneys, and common carotid artery (CCA) were harvested from rats. Tissues were fixed in 4% paraformaldehyde (PFA) for 48 h at 4 °C, dehydrated through a graded ethanol series (70%–100%), cleared in xylene, and embedded in paraffin. Sections (5 µm thickness) were cut using a rotary microtome (Leica RM2235), mounted on glass slides, and stained with hematoxylin and eosin (H&E). Histopathological analysis was performed under a bright‐field microscope (Olympus BX53), focusing on inflammatory infiltration, fibrosis, and structural integrity.

### Immunofluorescence Staining of Brain and Vasculature

For large vessel and neurovascular unit characterization, rats were transcardially perfused with ice‐cold PBS followed by 4% PFA under deep anesthesia. Brains were postfixed overnight in 4% PFA, cryoprotected in 30% sucrose solution, and sectioned coronally (10 µm thickness) using a freezing microtomecryostat (Leica CM1950). Antigen retrieval was performed in citrate buffer (pH 6.0, 95 °C, 20 min). Sections were blocked with 5% donkey serum in 0.3% Triton X‐100/PBS for 1 h at room temperature (RT), then incubated overnight at 4 °C with primary antibodies, including CD31 (vascular endothelial cells, Servicebio GB120005), α‐SMA (smooth muscle cells Servicebio GB111364), CD68 (macrophages, Servicebio GB113109), MAP2 (neurons, Servicebio GB11128), and GFAP (astrocytes, Servicebio GB15096). After PBS washes, sections were incubated with corresponding secondary antibodies for 2 h at RT. Nuclei were counterstained with DAPI (1 µg mL^−1^, Sigma‐Aldrich). Fluorescent images were acquired using a confocal laser scanning microscope (CLSM, Zeiss LSM 980) with consistent acquisition settings across groups.

### Mo Braided Stent Wire Implantation in tMCAO Rat for Safety Analysis–Surgery for Stroke Model and Mo Wire Implantation

The process until the exposure of right CCA, ICA, and ECA was the same as the previously mentioned procedure for Mo wire implantation surgery. Then, a thread was inserted from the tiny incision on the ECA to the ICA until reaching the opening of the middle cerebral artery (MCA) to occlude the MCA.^[^ ^47^ ^]^ After 2 h, the thread was removed and a Mo wire was inserted through the same incision on the ECA to the CCA and the wire was fixed on the ECA. Rats undergone MCA occlusion (MCAO) procedure and Mo wire implantation were set as tMCAO+Mo wire group and those undergone simple MCA occlusion procedure were set as tMCAO group. Rats undergone simple vascular separation surgery were set as sham group.

### Behavioral Test

Behavioral test included Longa score,^[^ ^47^ ^]^ rotarod test,^[^ ^48^, ^49^ ^]^ adhesion removal test,^[^ ^50^ ^]^ and open field test,^[^ ^51^ ^]^ which was conducted by an independent, blinded evaluator. Longa score: score criteria were defined as 0, no deficiencies; 1, incapacity to fully extend the contralateral forelimb; 2, rotating to the opposite side; 3, falling to the opposite side when walking; 4, diminished awareness and incapacity to walk. Rotarod test: rats were placed on a rotarod and its speed was increased from 4 to 40 rpm within 5 min. The time when rats fell off the rotarod is recorded. Adhesion removal test: adhesive tapes of equal specifications were used to cover the hairless area of the rats' left forepaws. It was recorded how long it took the rats to contact and pull off the tapes. If the rats failed to react within 2 min, it was recorded as 2 min. Open field test: rats were placed in a 100 cm × 100 cm × 50 cm cubic box and given 5 min to roam around. The moving trace was recorded and the distance of movement and duration of stay in different regions are calculated.

### Triphenyltetrazolium Chloride Stain

Rats were given phosphate‐buffered saline (PBS) intracardially for blood flushing, and their brains were taken out. After that, the brains were cut into 2‐mm coronal slices and stained for 20 min at 37 °C in the dark using 2% Triphenyltetrazolium chloride (TTC) reagent. Each section's infarct volume was determined using ImageJ software after it was photographed (National Institutes of Health, USA). Normal tissue was red, while the infarct area was white. Infarct volume (%) = (contralateral size – ipsilateral noninfarct size)/contralateral size × 100% was the formula used to determine the infarct volume.

### Nissl Stain

To assess neuronal death and survival, Nissl staining was conducted. The tissue was preserved for 24 h at 4 °C in 4% paraformaldehyde (PFA) (Biosharp, China). Nissl‐stained pictures were taken after sagittal brain sections (10 µm) were placed on slides and stained using the Nissl staining technique. Within the region of interest, five regions were chosen at random. On both hemispheres, the chosen region is perfectly symmetrical. It was determined how many Nissl‐positive cells were found in the ipsilateral hemisphere compared to the contralateral hemisphere.

### Evans Blue Extravasation Assay

Evans blue (EB) extravasation was performed according to the previously reported method.^[^ ^52^, ^53^ ^]^ Rats received an intravenous injection of 2% EB solution (wt/vol in PBS, 3 mL kg^−1^) 2 h prior to sacrifice. The rats' right ventricle was perfused with PBS in order to eliminate the intravascular EB solution. Then, the brains were removed and captured on camera. Next, PBS was used to cleanse and homogenize the brains. After an overnight incubation and shaking period at 60 °C in formamide, the EB was separated from the homogenates. Following 10 min of centrifugation at 5000 rpm, wavelength spectrophotometric analysis at 620 nm was used to measure the amount of EB present in the brain supernatants. The EB was serially diluted to create a standard curve. The standard curve was used to calculate the concentration of extravasated EB (µg of EB per g brain).

### Brain Water Assay

Weighing fresh brain tissue yielded its wet weight, which was subsequently dried in an oven to determine its dry weight. (Wet weight – dry weight)/wet weight × 100% was the formula used to determine the proportion of water content in brain tissue.^[^ ^54^ ^]^

### Mo Coil Implantation in Aneurysm Rat Model for Safety Analysis—Surgery for ECA Aneurysm Model and Mo Coil Implantation

In order to make the aneurysm model closer to the in vivo situation, that is, the loss of the middle layer elastic membrane in the arteries, the rat ECA aneurysm model based on previous research was optimized.^[^ ^55^, ^56^ ^]^ Briefly, as mentioned earlier, right CCA, ICA, and ECA was exposed. Then, the distal 6–7 mm to the origin of the ECA was ligated using a 6‐0 silk suture, and the origin of the ECA was temporarily blocked through a slipknot. Next, puncture is performed at the position slightly proximal to the distal ligation site using a 26G indwelling needle and secured with suture to prevent fluid from flowing out. Subsequently, protease (E1250, Sigma‐Aldrich, USA) was infused into the blood vessels through an indwelling catheter to fill the arteries, and the protease was retained in the artery for 10 min to fully digest the elastic membrane layer inside the artery. Last, the puncture site is closed with a thread and the slipknot at the origin of the ECA was opened to recanalize the blood flow to ECA, and the ECA was transected just distal to the puncture site. The wound of the rat was closed in multiple layers with 4–0 suture, and the rat was put back into cage with adequate water and food.

Three days later, after the arterial aneurysm model stabilized, the right ECA aneurysm model, CCA and ICA were re exposed after anesthesia in rats. Then, ICA and CCA are temporarily blocked through slipknots. Next, at the top of the aneurysm model a small incision was made using microscissors. Later, 4–5 mm Mo coils were implanted in ECA aneurysm model to completely fill aneurysm through this incision which was subsequently sealed with sutures and the slipknots at ICA and CCA were opened. The wound was closed and the rat was put back into the cage. Rats receiving Mo coil implantation were assigned to the Mo coils group, while rats receiving the same procedure except for coil implantation were assigned to the control group.

### Laser Speckle Imaging for ECA Aneurysm Model

Laser speckle imaging (LSI) was used to visually display the blood flow inside the aneurysm before and after the implantation of the Mo coils. Before implanting the coil, the rat was placed in a supine position under the LSI device (RWD Science Co., China), and the positions of ECA aneurysm, CCA, ICA were adjusted to clearly image the blood flow in them. Then, the intravascular blood flow immediately after the implantation of the Mo coils and 1 month after implantation was also detected under the same procedure.

### Digital Subtraction Angiography for ECA Aneurysm Model

At 1 month after the operation, an angiography was done through left CCA. A 20G indwelling needle was inserted and fixed into the left CCA of anesthetized rats that had been put supine on an X‐ray compatible table. Through the indwelling needle, 150 IU kg^−1^ of heparin diluted in normal saline was given to avoid blood clotting during the operation. In order to opacify the arteries and the aneurysm sac, 2.5 mL of contrast media was injected through an indwelling needle under a digital subtraction angiography (DSA) system (Philips, the Netherlands).

### In Vivo Mo Coil Degradation

In order to detect the degradation process of the Mo coils in vivo, an operation was performed similar to Mo wire degradation detection, but limited the collected specimens to blood and organs, and perform the same operation on them for ICP detection.

### Histological Evaluation of Organs and ECA Aneurysm

The histological evaluation of organs of rats implanted with Mo coil was same as those implanted with Mo wire, which was described above. For histological evaluation of Mo coil implanted ECA aneurysm, the aneurysm tissue samples were extracted and preserved for 48 h at RT in paraformaldehyde. A photocuring embedding machine (EXAKT 520, Exakt, Germany) was then used to embed the specimens in methyl methacrylate after they had been dried in a water extractor (EXAKT 510, Exakt, Germany) and cleared in acetone. Then, the aneurysm specimens were transversely cut and grounded into 20 µm slices (EXAKT 300CP and 400CS, Exakt, Germany). Last, H&E were used to stain the slices which was further photographed for analysis.

## Conflict of Interest

The authors declare no conflict of interest.

## Supporting information



Supporting Information

## Data Availability

The data that support the findings of this study are available from the corresponding author upon reasonable request.
