# Supplementary material for: Ultrafine Molybdenum Wire Braided Neurointerventional Implants: Bridging Biodegradability and Neurovascular Safety for Stroke Treatment
Source: Adv Sci (Weinh). 2025 Sep 29;13(1):e11466. doi: 10.1002/advs.202511466 (PMC12767060; doi:10.1002/advs.202511466)
Supplement: Supplementary file 1 — Supporting Information [file ADVS-13-e11466-s001.docx]

**Figs**


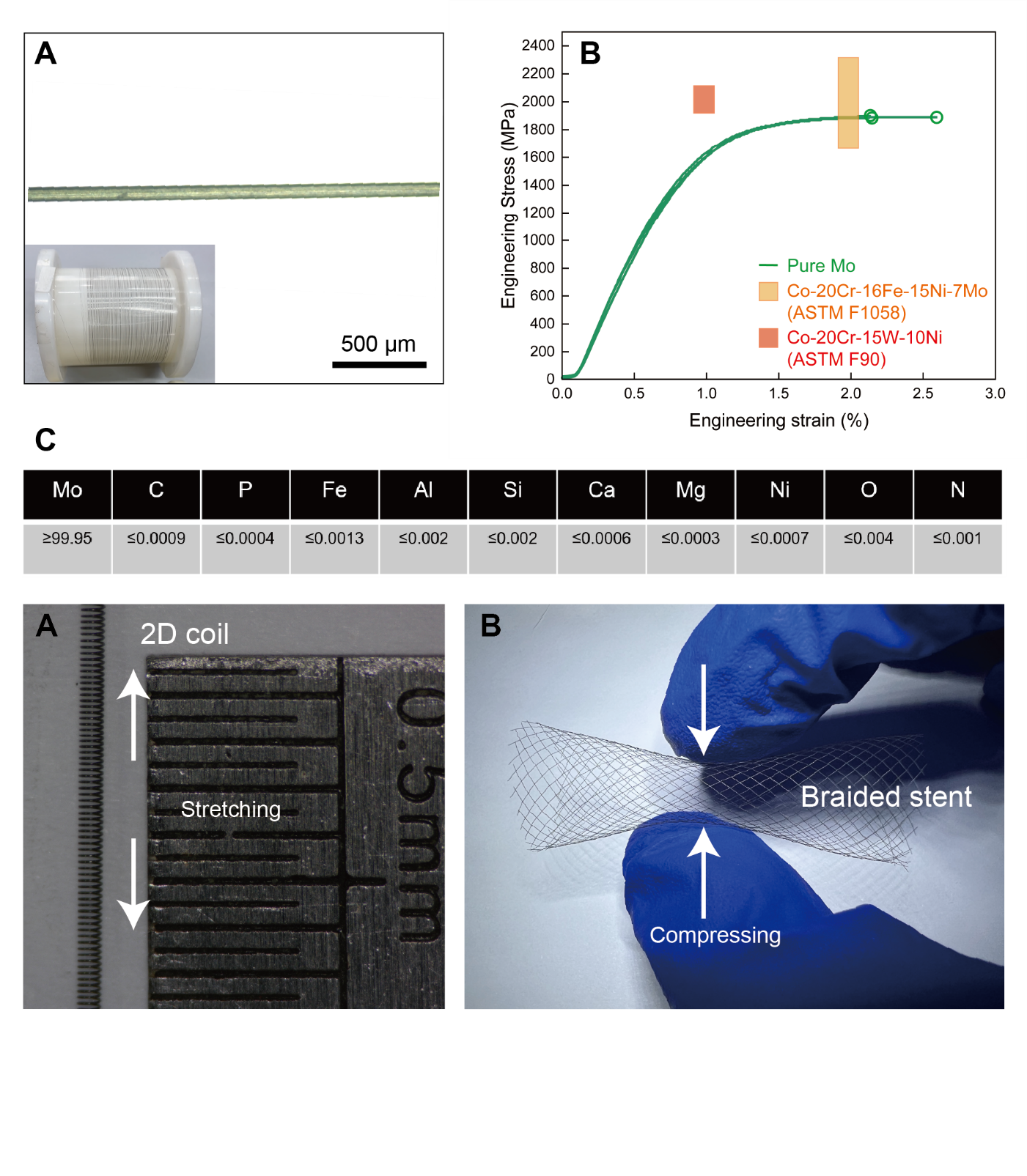


**Fig. S1** Photos of (A) 2D Mo coil & (A) braided Mo stent


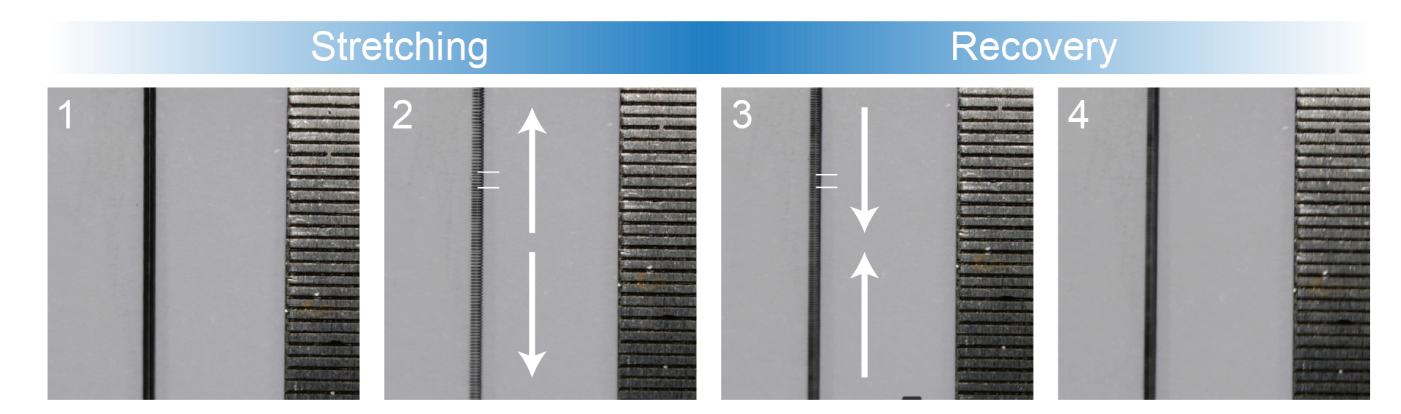


**Fig. S2** Deformation and shape recovery process of 2D coils under tensile loading


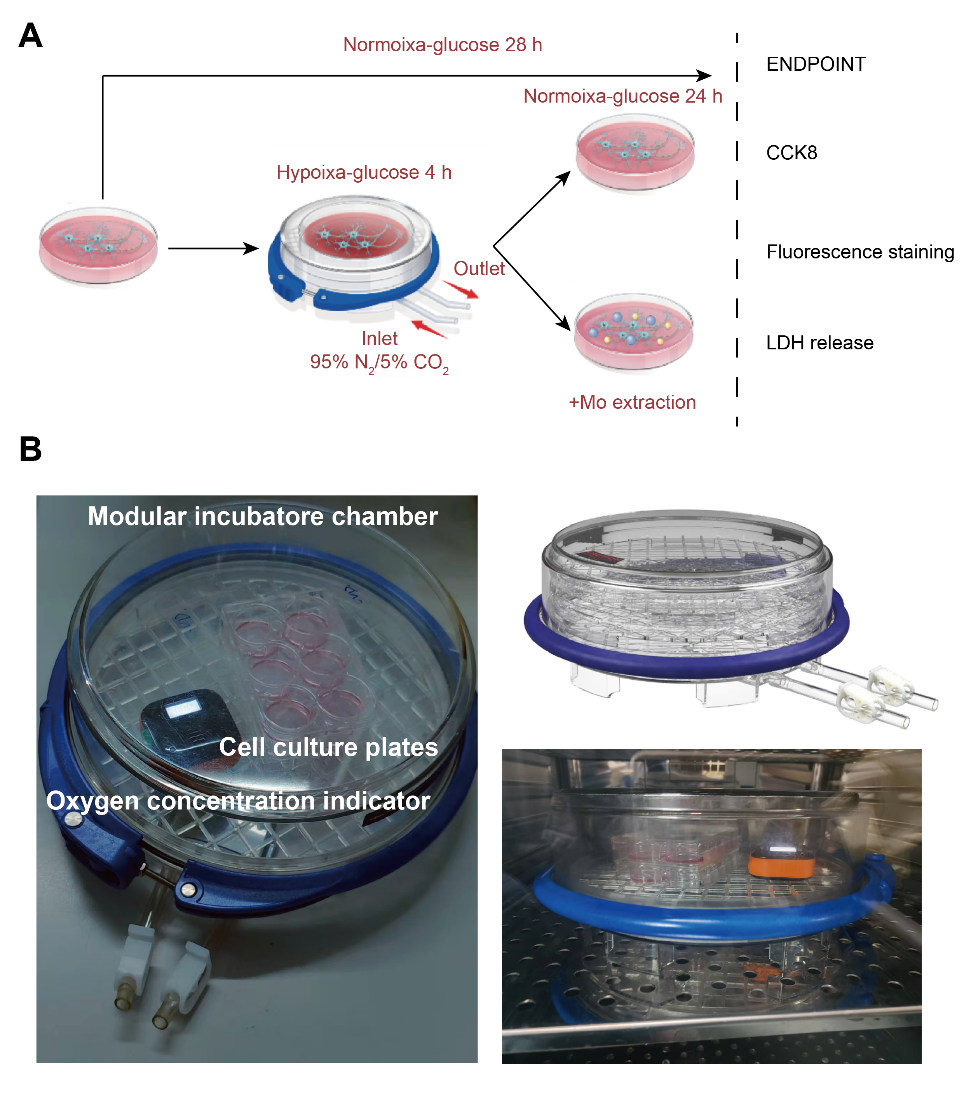


**Fig. S3** (A) Schematic diagram of the OGD/R model operation. (B) Photographs of the OGD/R model operation.


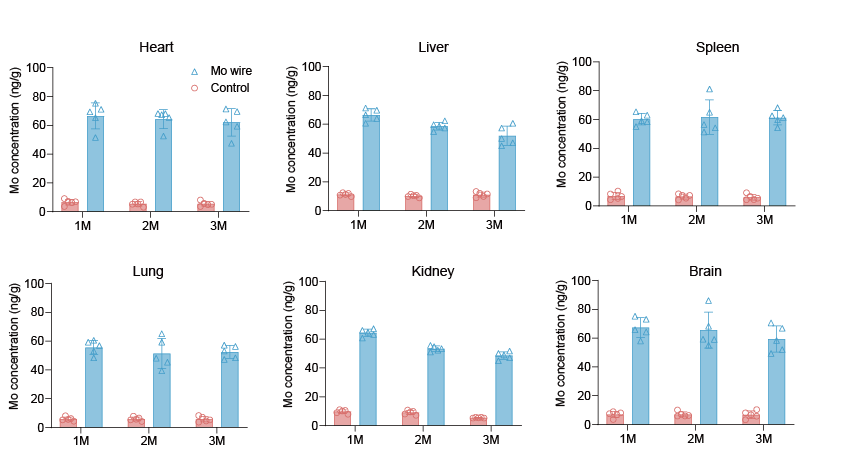


**Fig. S4** Mo ion concentration in organs of rats with CCA-implanted wires and control groups at 1, 2, and 3 Months


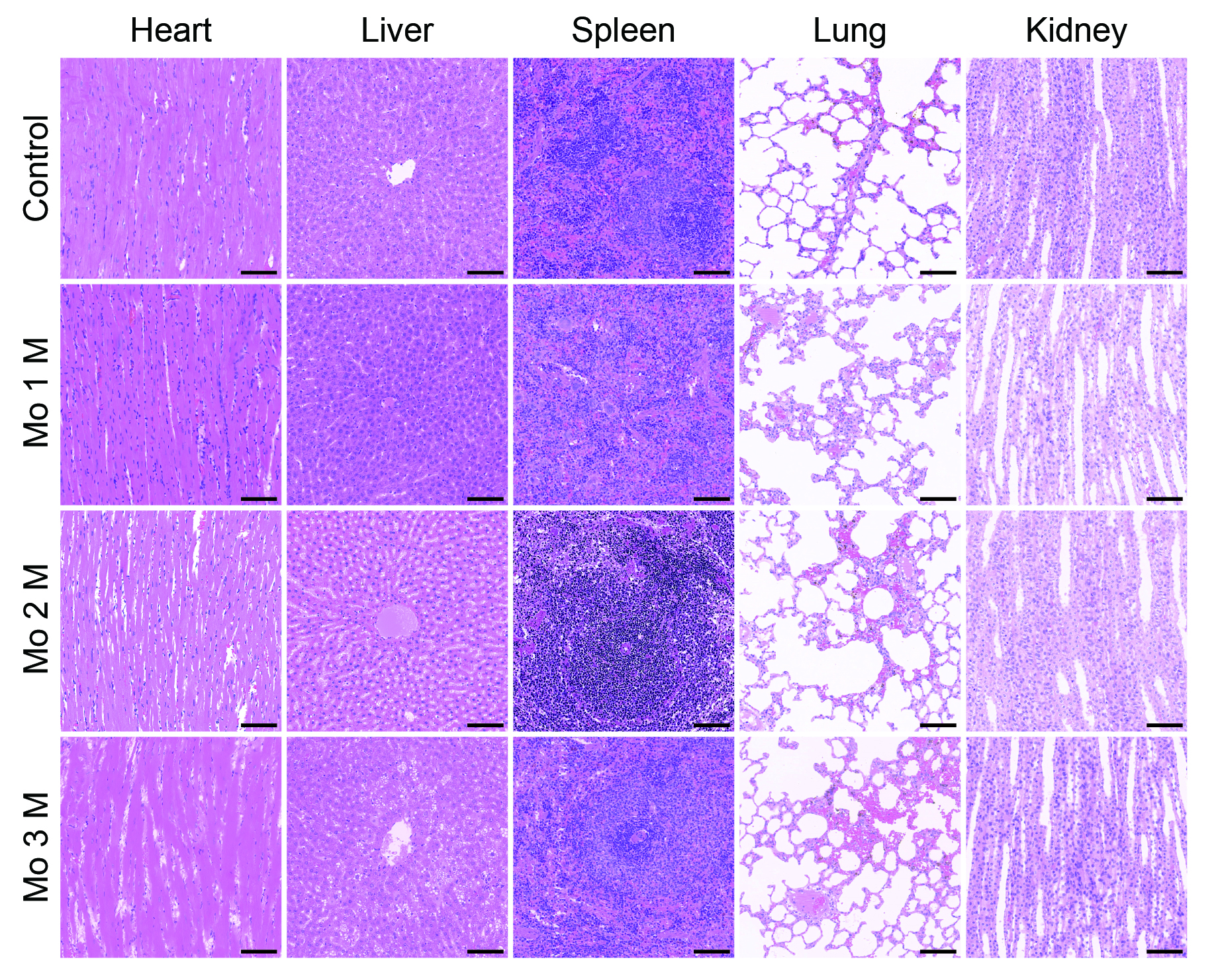


**Fig. S5** H&E-stained tissue sections (heart, liver, spleen, lung, kidney) from SD rats after 1-, 2- and 3-months implantation of Mo wires and SD rats with no implantation as a control.


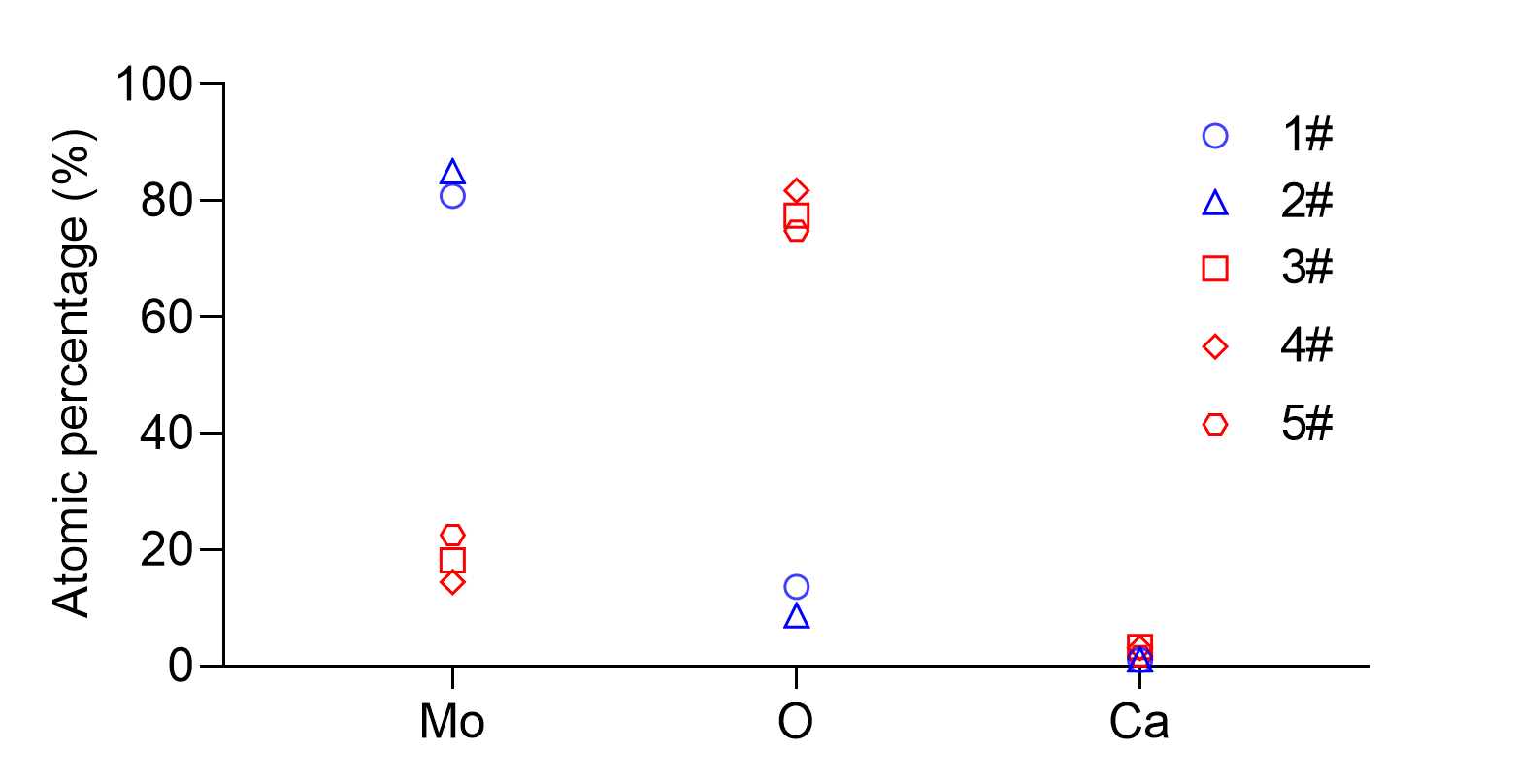


**Fig. S6** EDS analysis of regions 1-5 marked in Figure 4P, showing the elemental composition of the Mo wire after 3-month vascular implantation


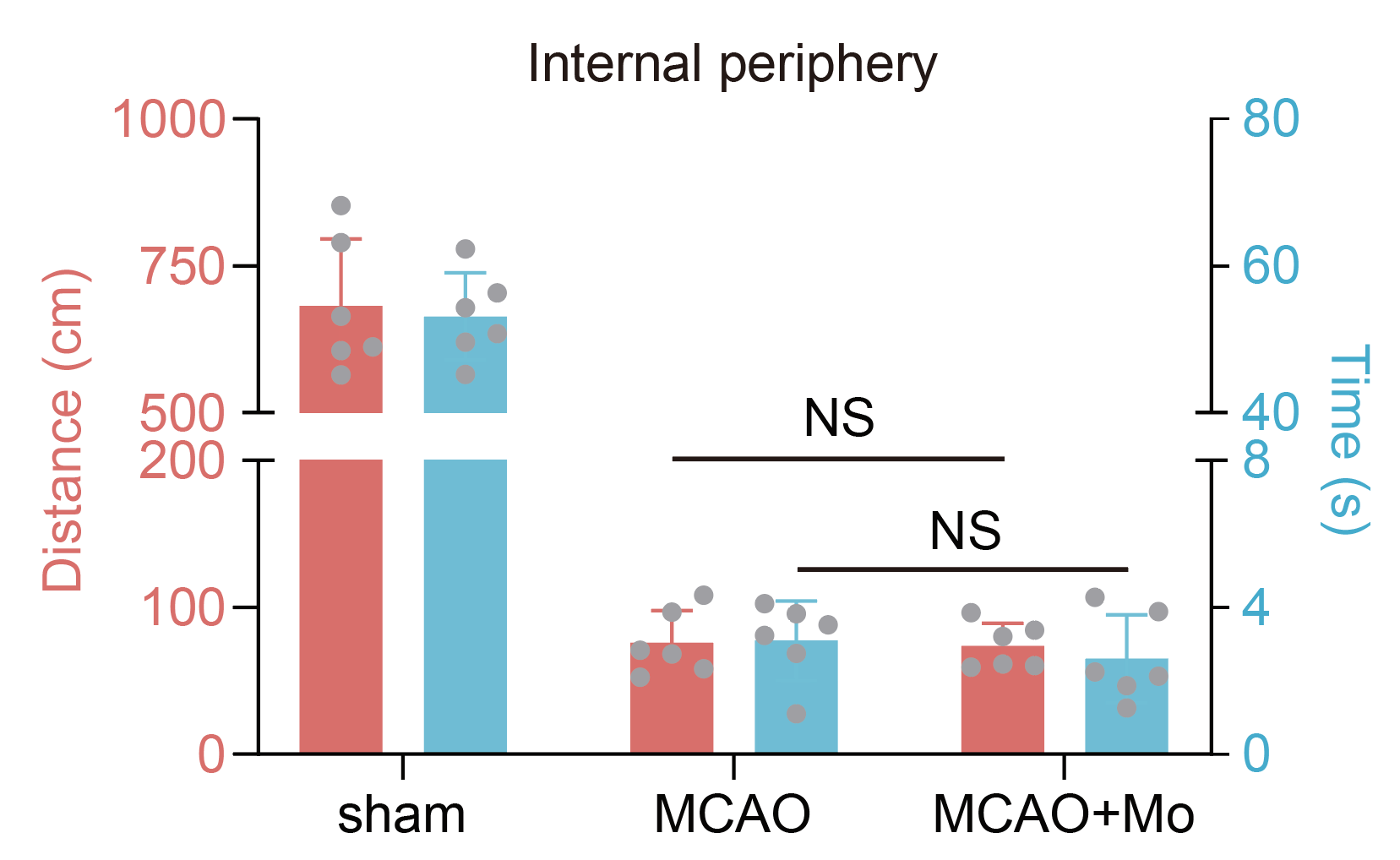


**Fig. S7** Open field test. NS, not significant.


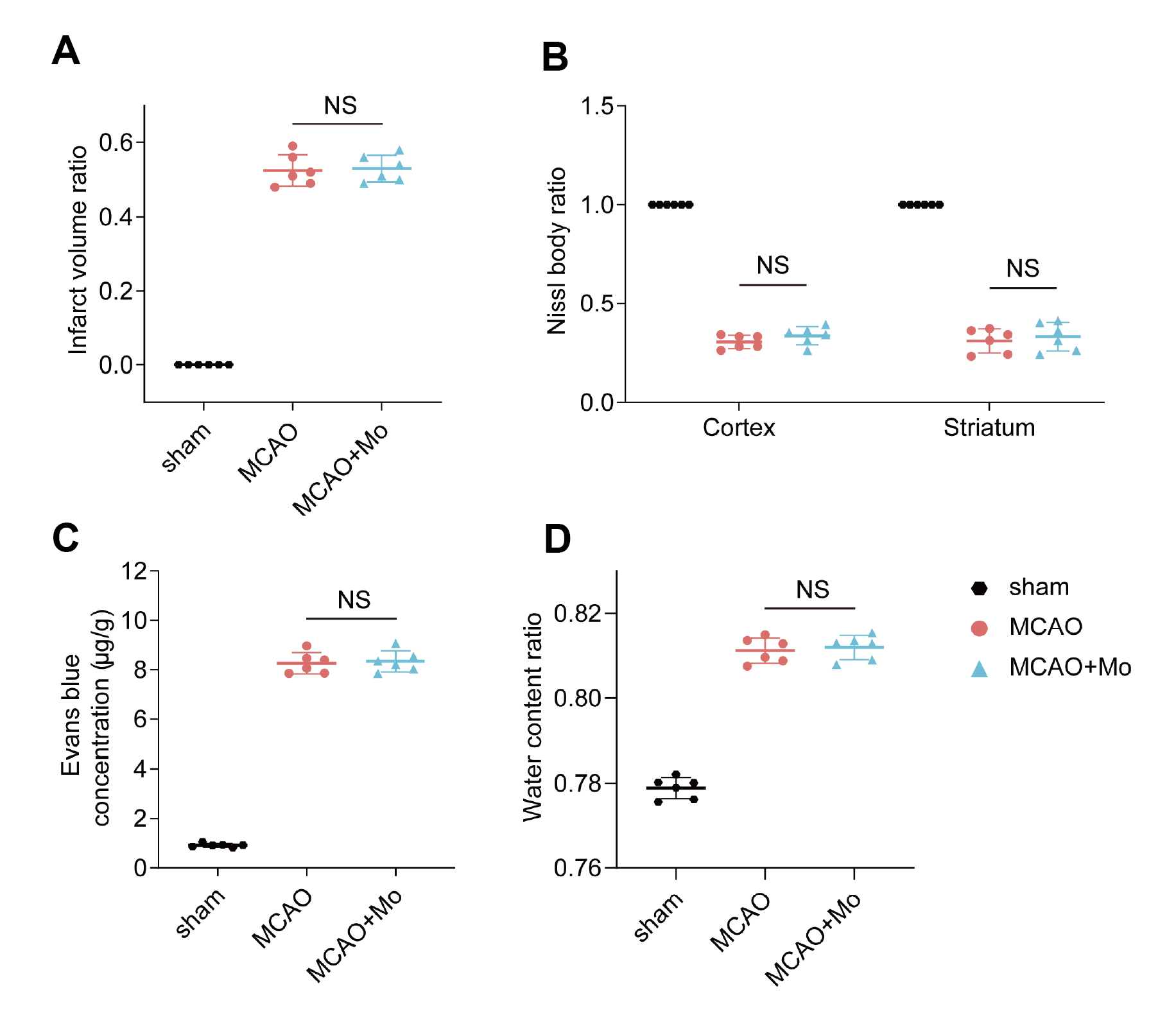


**Fig. S8 In vivo brain integrity analysis of Mo wires implantation after ischemia/reperfusion injury.** (A) Quantification of brain infarct volume from. (B) Quantification of Nissl bodies. (C) Quantification of cerebral Evans blue content. (D) Evaluation of brain water content ratio. NS, not significant.


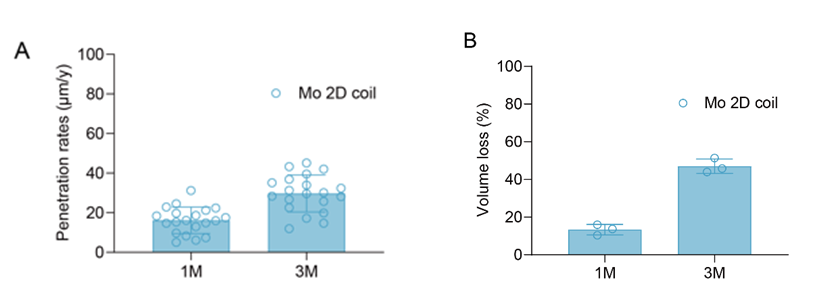


**Fig. S9** Quantitative analysis of penetration rates and volume loss in 2D coils


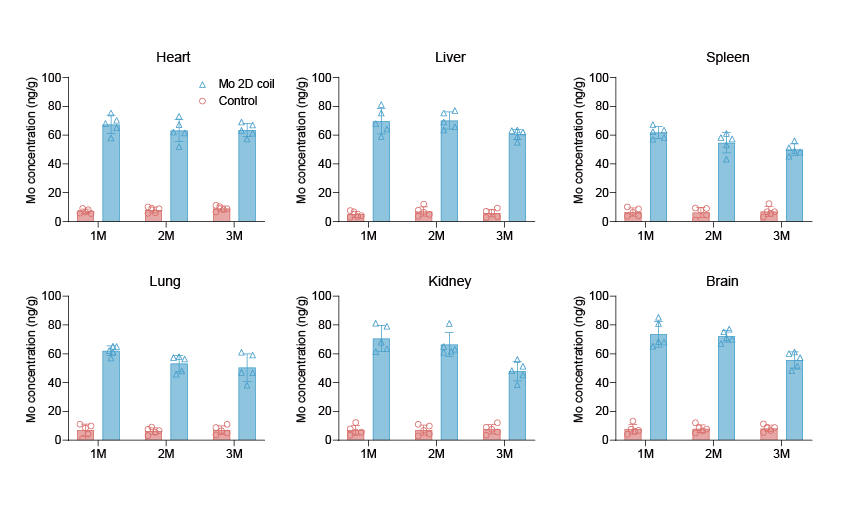


**Fig. S10** Mo ion concentration in organs of rats with aneurysm-implanted 2D coils and controls at 1-, 2-, and 3-months post-implantation


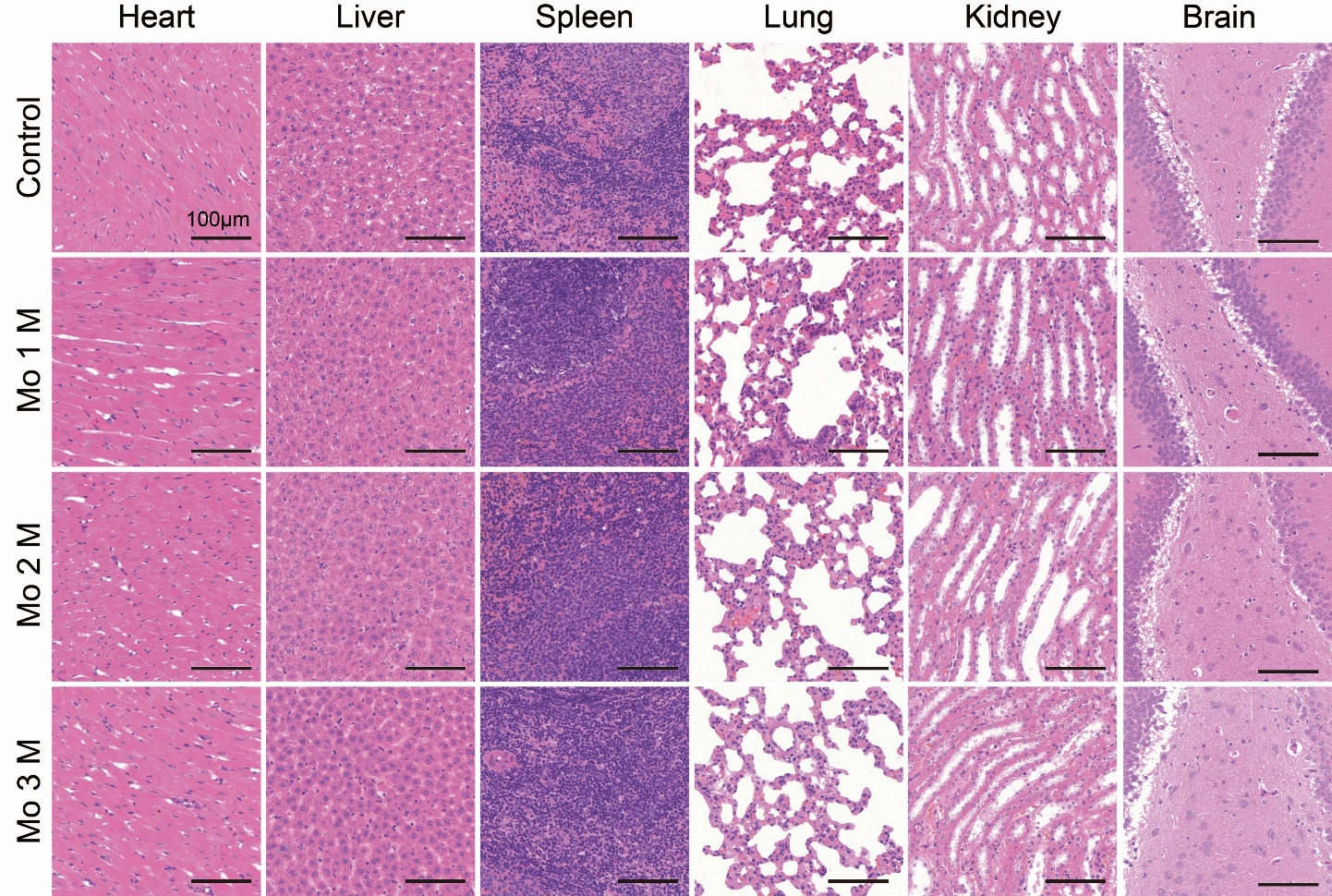


**Fig. S11** H&E-stained tissue sections (heart, liver, spleen, lung, kidney and brain) from SD rats after 1-, 2- and 3-months implantation of Mo coils and SD rats with no implantation as a control.


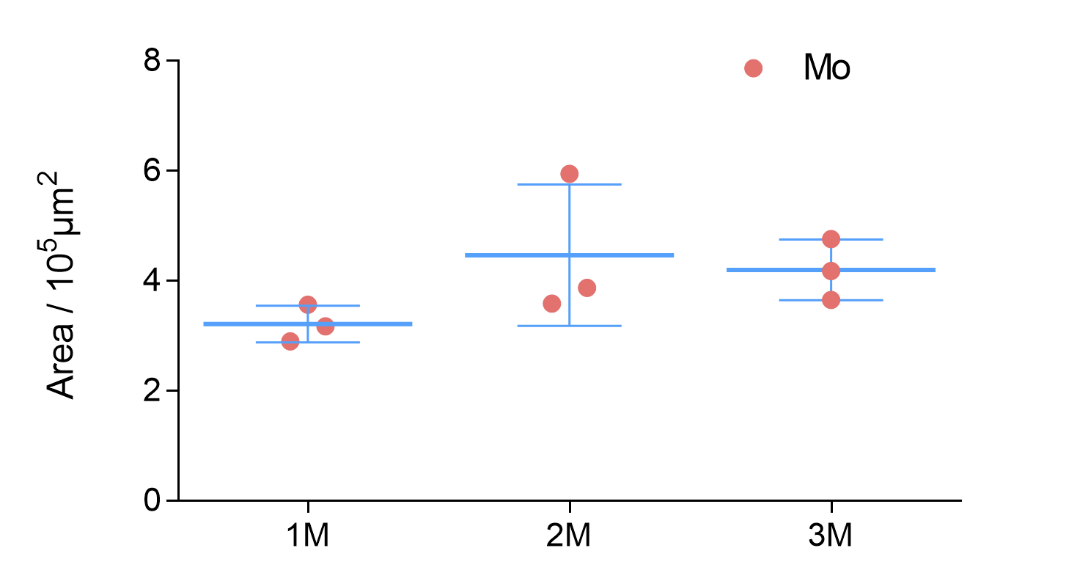


**Fig. S12** Quantification of neotissue area within 2D coils


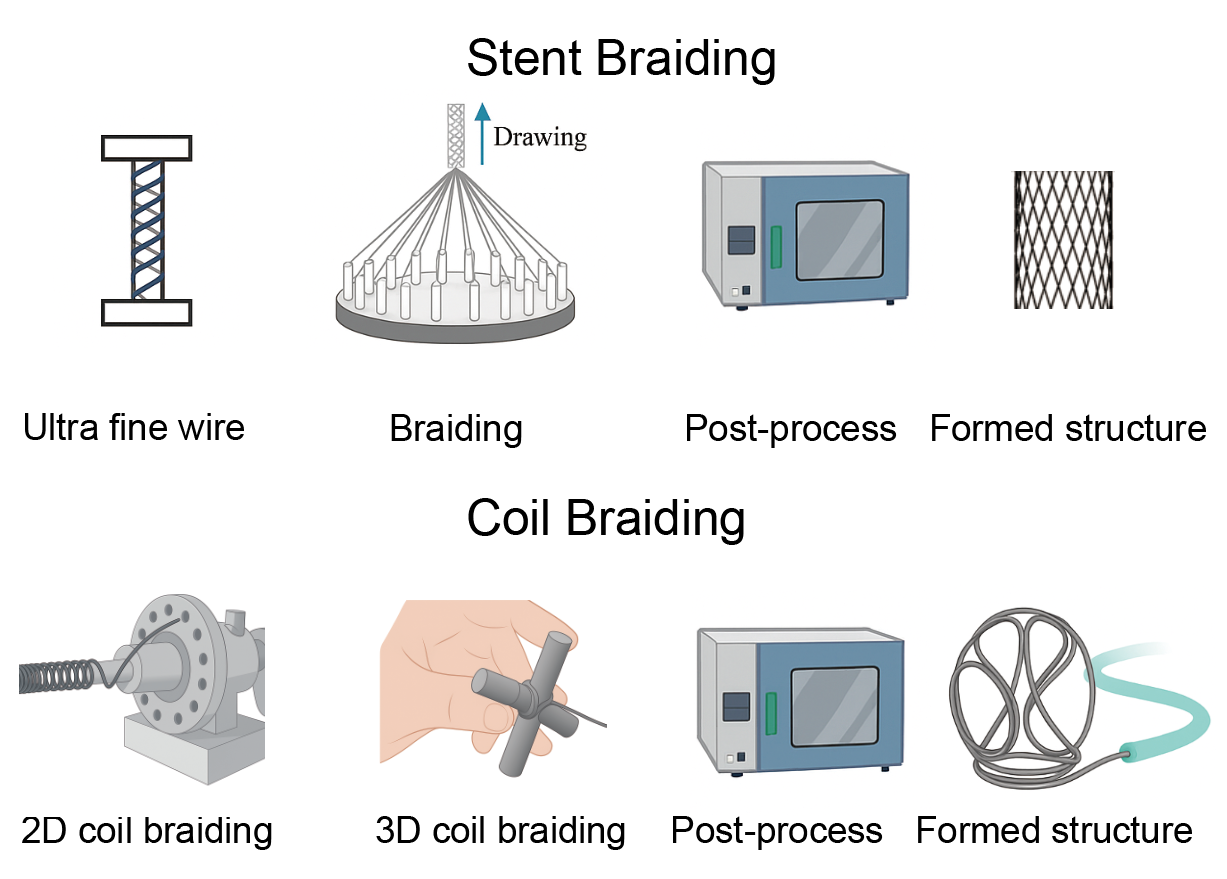


**Fig. S13** The fabrication process of the Mo stent and Mo coil
